# The structure of resilience in irritable bowel syndrome and its improvement through hypnotherapy: Cross-sectional and prospective longitudinal data

**DOI:** 10.1371/journal.pone.0202538

**Published:** 2018-11-12

**Authors:** Johannes Peter, Ulrich S. Tran, Maria Michalski, Gabriele Moser

**Affiliations:** 1 Gastroenterology and Hepatology Division, Department of Internal Medicine III, Medical University of Vienna, Vienna, Austria; 2 Department of Basic Psychological Research and Research Methods, School of Psychology, University of Vienna, Vienna, Austria; Public Library of Science, UNITED KINGDOM

## Abstract

**Background:**

Resilience refers to a class of variables that are highly relevant to wellbeing and coping with stress, trauma, and chronic adversity. Despite its significance for health, resilience suffers from poor conceptual integration. Irritable bowel syndrome (IBS) is a functional disorder with altered psychological stress reactivity and a brain-gut-microbiota axis, which causes high levels of chronic strain. Gut-directed Hypnotherapy (GHT) is a standardized treatment for IBS aimed at improving resilience. An improvement of resilience as a result of GHT has been hypothesized but requires further investigation. The aims of the study were to validate the construct and develop an integrational measure of various resilience domains by dimensional reduction, and to investigate changes in resilience in IBS patients after GHT.

**Method:**

A total of *N* = 74 gastroenterology outpatients with IBS (Rome III criteria) were examined in 7 resilience domains, quality of life, psychological distress and symptom severity. Of these, *n* = 53 participated in 7 to 10 GHT group sessions (Manchester protocol). Post-treatment examinations were performed on average 10 months after last GHT session.

**Results:**

Resilience factors proved to be unidimensional in the total sample. Greater resilience (composite score of resilience domains) and quality of life, and lower symptom severity and psychological distress were found after treatment (*n* = 16). Similar differences were present in cross-sectional comparisons of *n* = 37 treated vs. *n* = 37 untreated patients.

**Conclusion:**

Resilience factors share a common psychological dimension and are functionally connected. The absence of maladaptive behaviours contributes to resilience. Improvements in resilience after hypnotherapy with parallel increases in quality of life and reduced psychological distress and symptom severity were observed. Independent replications with larger sample sizes and randomized controlled trials are needed.

## Introduction

### Resilience

The term resilience refers to a class of psychological and behavioural variables essential to adaptive and coping ability. These variables are indicative of an individual’s capacity to maintain or regain well-being and psychological equilibrium in the face of stress, trauma, or chronic adversity, and are crucially connected to processes of mental health promotion, protection and recovery. Davydov et al. [1] drew an analogy between resilience and somatic models of immunity in medicine. However, the construct still needs conceptual progress, especially regarding the integration of different proposed factors and their psychometric measurement. There is noticeable overlap between resilience domains derived from different lines of research (Table 1); for overviews see [1, 2].

**Table 1:** Proposed psychological and behavioural factors of resilience.

Optimism and adaptive explanatory style [3, 4]
Active coping style [5, 6], and physical activity [7, 8]
Positive emotionality [9], humour [10], emotion regulation and reappraisal ability [11]
Self-efficacy [12] and coping ability appraisals [13]
Social support [14] and attachment security [15]
Purpose and value orientation [16], spirituality [17] and mindfulness [18]
Cognitive [19] and executive functioning [20]

The familiarity and functional relatedness between different resilience domains is obvious. This may represent the inherent qualities of resilience variables themselves, which is a subject of investigation in this study. But it is also caused by a multitude of similar constructs and different operationalizations used in the field. Resilience research suffers from poor concept definition and the lack of a unified methodology [1, 21]. An integrative framework for the study of resilience that emphasizes the role of stimulus-appraisal processes in response to stressors has been proposed [22]. Progress has also been made in the investigation of neurobiological aspects of resilience [23, 24]. Further effort in integrating behavioural and psychological mechanisms also seems necessary to overcome the fragmentation of resilience and its related constructs, and to facilitate the design of interventions for promotion of mental health and well-being. A meta-analysis of interventions aiming to improve resilience has shown small to moderate effects [21].

### Seeing both sides of the coin: Positive psychology and absence of pathology

Resilience research is profoundly linked with positive psychology, and focusing on resources and strengths instead of on pathology represents a paradigmatic shift in psychology and health professions [25]. However, exclusively taking account of the ‘positive’ side neglects an important fact: well-being and adaptation are supported not only by the presence of resources and positive behaviours, but also by the *absence* of detrimental behaviours. Maladaptive emotion-regulation strategies [11], perceptions of negative affect, and emotional instability are related to negative developmental trajectories. They are also linked to the personality dimension of neuroticism [26]. In the conceptual framework by Kalisch et al. [22], this aspect is reflected in the axiomatic mechanism of interference inhibition: inhibition of mentally costly negative emotional reactions is considered to be crucial for adaptation to stress and to positive health outcomes.

### Irritable bowel syndrome

Chronic diseases cause great psychological strain. Coping, adapting, and resilience are therefore of great significance in chronic physical illness and psychosomatic conditions [27]. Irritable bowel syndrome (IBS), a functional disorder affecting the gastrointestinal tract, is a condition that leads to significantly diminished quality of life [28]. Prior research has provided evidence of reduced resilience in IBS, compared to healthy controls [29, 30], and of the important role of resilience, cognitive appraisal, and coping styles for health-related outcomes in IBS [31, 32].

IBS is characterized by altered bowel habits and perceptions of pain, discomfort, and concomitant distress. Its pathophysiology is described as an imbalance in the *brain-gut axis* with perturbations of visceral homeostasis, exaggerated autonomic reactions and hyperalgesia. It also includes a number of psychological mechanisms such as hypervigilance towards interoceptive signals, alexithymia [33] and enhanced responsiveness to psychosocial stressors [34, 35]. The majority of IBS patients report emotional problems, such as anxiety and depression [36], which suggests that psychological changes are a crucial element in the disturbed brain-gut communication in IBS [37]. Gut microbiota changes may also be an important factor in IBS pathophysiology. Relatively recent advances in this field point to a connection between gut microbiota and visceral hypersensitivity, immune functioning and neurotransmitter metabolism, but also with behavioural patterns of anxiety and depression, stress reactivity and resilience [38, 39].

Reduced healthcare consumption following a relaxation-response-based resilience intervention has been reported in one controlled cohort study [40]. These findings are relevant to the treatment of IBS, since its economic impact is known to be severe [41].

### Gut-directed hypnotherapy and its central nervous impact

Psychological needs are recognized to be an important management issue in IBS [42] and have entered clinical guidelines [43]. A number of psychological therapies for IBS have been developed [44], among which, gut-directed hypnotherapy (GHT) is a prominent and well-researched therapy [45, 46]. There is evidence for several pathways of action of GHT, ranging from the immunological impact (albeit ascertained only in inflammatory bowel disease patients so far) [47], effects on gut motility [48] and on autonomic nervous system activity [49], to a reduction of visceral hypersensitivity [50]. However, these effects have not been consistently reported and are rather limited in relation to the magnitude of the therapeutic effects observed. Consequently, the assumption has been made that GHT primarily acts by its effect on central processing [49, 51]. This is underpinned by a study that showed that dysfunctional gastrointestinal-related cognitions decrease with GHT [52], and by a neuroimaging study that found altered functional cerebral connectivity reflecting normalized processing of visceral stimuli after GHT [53].

Led by the observation that many patients without direct symptom improvements report high levels of satisfaction with this therapy and an improved quality of life, Lindfors et al. [54] were the first to posit that GHT might benefit coping. However, in their trial [54] sense of coherence [55], a construct considered inclusive of resilience [56], did not improve significantly after GHT. Nevertheless, the assumption of an improvement in resilience as a result of GHT is compelling: the treatment protocol contains suggestions targeting perceptions of control, problem-solving ability, and positive self-esteem. Furthermore, the experience of relaxation and positive body experiences (e.g., warmth by application of the patients’ own hands) and suggestions of self-healing may strengthen the belief in self-regulative and self-healing ability. We hypothesized that it is precisely this that affects the class of variables linked to resilience (Table 2).

**Table 2:** How gut-directed hypnotherapy might enhance resilience and coping.

**Suggestions targeting:**
Emotional security
Reduction of dysfunctional cognition
Self-efficacy
Control over bodily processes
Sense of self-healing
**Direct experience of:**
Relaxation
Positive body experience
Inner resources (e.g., pleasant memories)
Positive emotion
Effective self-regulation

### Aims of the present study

The perspective of this study is observational and originates from the clinical practice at a tertiary care centre. Within the context and limitations of this setting, the study aimed (1) to assess factors of resilience in a sample of IBS patients and to investigate whether they were facets of the same underlying construct; (2) to calculate one or more composite scores for resilience that captured its latent factors; and finally, (3) to investigate differences between patients untreated or treated with GHT in groups by cross-sectional and longitudinal comparisons, testing the hypothesis of an improvement in resilience (using the composite score[s]), with changes in IBS symptoms, quality of life and psychological distress in parallel.

## Methods

### Study location and recruitment

The study was conducted at the specialist outpatient-clinic for psychosomatics at the Gastroenterology and Hepatology Division, Department for Internal Medicine III, University Hospital of Vienna.

Included were IBS patients diagnosed according to Rome III criteria, aged between 18 and 75, and refractory to other IBS therapies. ‘Refractory’ here refers to IBS patients who had failed to improve on a variety of therapies (IBS medications, antidepressants, probiotics, psychotherapy), who were unhappy about their care and who had a persistently high rate of healthcare consumption [57]. Patients with acute medical complications, pregnancy or insufficient knowledge of German were excluded from this study.

Data collection was performed in two stages. Patients in Group A had participated in prospective routine assessments before undergoing gut-directed hypnotherapy treatment prior to the current study. GHT is a routine treatment at the study site and had therefore been administered to the patients of Group A before they also gave their consent to participate in the current study. Patients in Group A were repeatedly contacted via telephone and/or mail to participate in this study, which involved participation in a follow-up assessment and read-out of existing routine data. Patient information, informed consent form and questionnaires for participation in the current study were sent via mail, also including a stamped-addressed envelope for returning the completed documents to the hospital.

Patients in Group B were recruited directly at the outpatient clinic, consecutively as referred and as potential candidates for GHT. A subset of patients in Group B were administered GHT after the baseline examination. These patients were followed-up the same way as patients in Group A. Antidepressants, anxiolytics and/or ongoing psychotherapy were allowed, since comorbid psychological diagnoses, a common problem in IBS patients, were present in the study sample. The study was registered at ClinicalTrials.gov (ID: NCT02737410; see S1 Appendix). Registration at ClinicalTrials.gov took place after enrolment started, because the study was reviewed and registered locally (approval by the ethics committee of the Medical University of Vienna on 4^th^ September 2012, ID: 1488/2012). The authors confirm that all ongoing and related trials for this intervention are registered. Each participant gave their written informed consent.

Calculations of sample size were based on data from a prior gut-directed hypnotherapy trial [58] at the study site and yielded a required group size of 49 subjects (see appendix for details). Due to limited research resources, recruitment aimed at only 40 patients per group.

### Study conduction and treatment

Patients in Group A were routinely assessed immediately before GHT and treated from January 2010 to June 2012. These patients were contacted and sent questionnaires to participate in a follow-up examination from October 2012 to January 2013. Overall, *n* = 67 patients in Group A were contacted via telephone and/or mail, of which *n* = 4 declined to participate, *n* = 2 were uncontactable due to unknown address, and *n* = 24 did not return the questionnaires. Specific reasons for non-participation were not disclosed and could not be ascertained (e.g., dissatisfaction with treatment, unattainability or for other reasons). No financial or other incentives were given for study participation or for returning the questionnaires. Completed questionnaires were returned by *n* = 37 patients in Group A.

**Fig 1:**
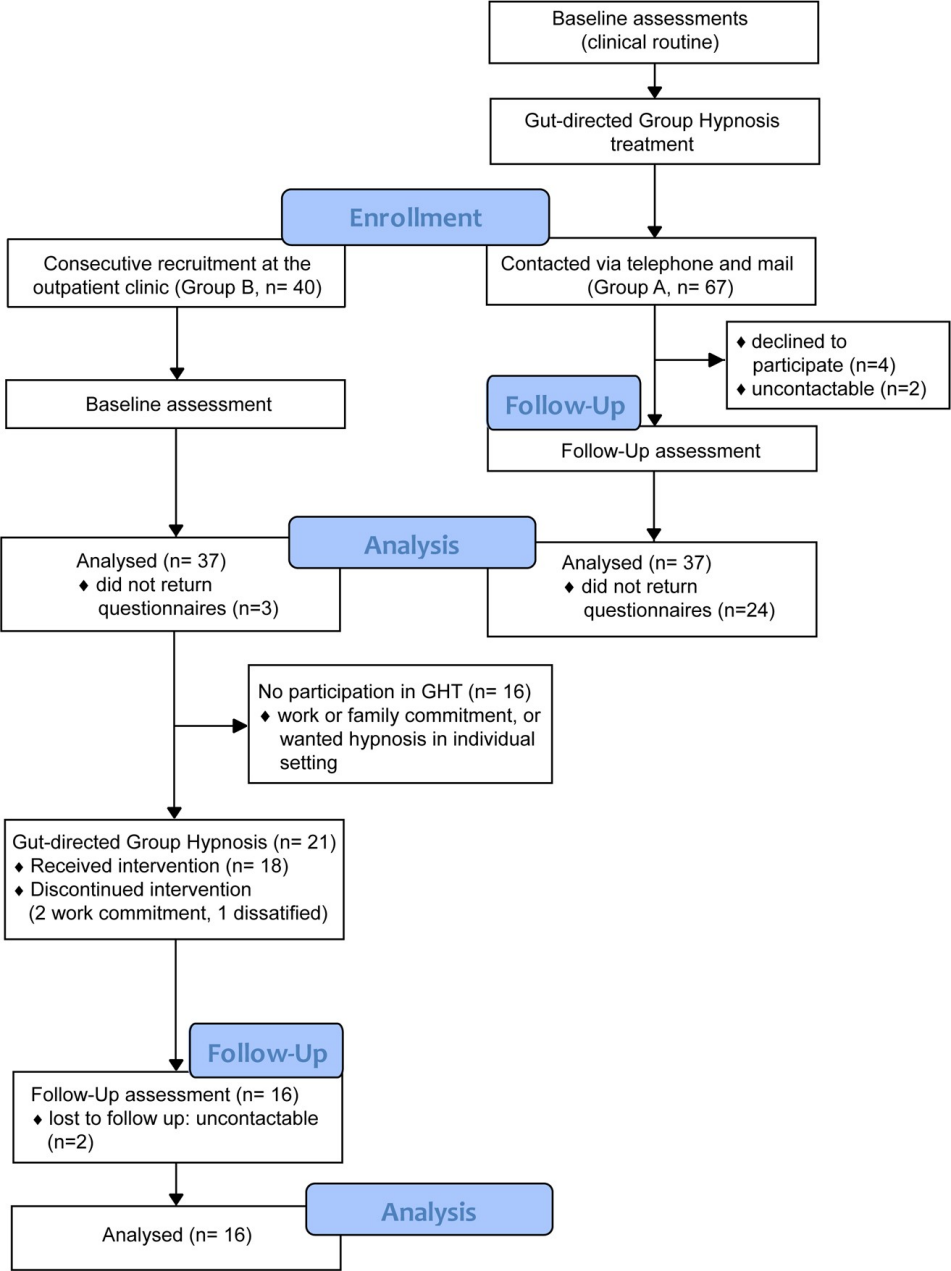
Patient flow diagram.

Patients in Group B (*n* = 40) were contacted directly and recruited consecutively at the outpatient clinic from October 2012 to March 2013. Three (*n* = 3) patients did not return questionnaires, resulting in *n* = 37 questionnaires in Group B. Organizational feasibility and willingness to participate in the group therapy was present in *n* = 21 patients in Group B. GHT was provided to these patients between November 2012 and October 2013; follow-up/post-treatment examinations were performed between January and May 2014. The follow-up examination of patients in Group B after treatment was not planned or indicated in the original study protocol (see S2 and S3 Appendix), but since there was a demand for therapy and willingness to participate in further examinations in the 21 patients, there was a follow-up to collect further longitudinal data, increasing the validity of the study. Of the patients treated in Group B, *n* = 5 were lost to follow-up (2 uncontactable, 2 work commitment, 1 dissatisfied; Fig 1). Post-treatment examinations were performed approximately 10 months after the last GHT session in both groups (*M*[ean] = 10.3 months after the last GHT session, range: 1–31 months in *n* = 37 of Group A; *M* = 10.4 months after last GHT session, range: 3–16 months in the subset of *n* = 16 patients treated of Group B). No adverse events were reported during or after the study.

There was no blinding of investigators or patients. The GHT treatment protocol used was the Manchester protocol of GHT [58, 59] and consisted of 10 weekly sessions (45 min) with six patients per group over a treatment period of 12 weeks. GHT was performed at the University Hospital by two experienced physicians (GM, MM) trained in Manchester (UK).

### Assessment

All scales were administered to participants in German. Previously published and validated German versions had been applied except for core resilience (10-item CD-RISC) and IBS symptom severity (IBS-SSS). In these cases translations by our own research group(s) were used. In both cases, translation into German, independent back-translation into English, and comparison of both English versions for equality by English native speakers, as appropriate, had taken place. Equivalent German translations (with validation data assuring psychometric validity) have been published in both cases in the interim.

#### Primary outcome: Resilience questionnaires

Resilience as the primary outcome variable(s) in this study was assessed with a broad measurement approach using several questionnaires capturing variables with a known connection to resilience (‘resilience factors’), which can be assigned to 7 domains.

‘*Core-resilience’* was assessed using a German form of the 10-item Connor-Davidson Resilience Scale (CD-RISC) [60], which is a short version of the original CD-RISC economically measuring an individual’s perceived ability to adapt to change and to cope with stress or hardship. It was rated as one of the better short instruments for assessing resilience [61]. The German version had been translated into German within a previous trial [60]. Responses are scored on a 4-point scale. Sarubin et al. [62] have published an equivalent translation and confirmed the unidimensionality of the German version. They reported an internal consistency of α = .84 [62].

S*elf-efficacy* was assessed using the *Skala zur allgemeinen Selbstwirksamkeitserwartung* (SWE) [63], a 10-item German scale on perceptions of self-efficacy as the ability to handle and cope with a broad range of challenging situations optimistically. Responses are scored on a 4-point scale. For the SWE, unidimensionality and an internal consistency of α = .92 are reported [64].

*Humor* was assessed using the trait cheerfulness subscale of the State-Trait Cheerfulness Inventory (STCI) [65], a 20-item scale assessing the three subdimensions of cheerfulness, seriousness, and bad mood as central dispositions for the experience of positive emotion through humour. Responses are scored on a 5-point scale. A validation study corroborates the psychometric quality of the scale [66].

S*ocial support* was recorded using the *Fragebogen zur sozialen Unterstützung Kurzform* (F-SozU K-14) [67], a 14-item German scale measuring social support as the subjective appraisal of support and access to resources received from the social environment at the present or in case of need. Responses are scored on a 5-point scale. The F-SozU K-14 is unidimensional, with a reported internal consistency of α = .94 [67].

*Emotion regulation* was assessed with the German form of the Cognitive Emotion Regulation Questionnaire (CERQ) [68], which records 9 strategies of emotion regulation as cognitive reactions to aversive events and their associated emotions. The strategies can be categorized into functional or dysfunctional strategies. *Positive reappraisal*, *positive refocusing*, *putting into perspective*, *acceptance* and *refocusing on planning* are considered functional; *catastrophizing*, *self-blame*, *rumination*, and *other-blame* are considered dysfunctional. Each regulation strategy is assessed under three items, making 27 items in total. It was suggested that the *rumination* subscale should be eliminated in order to improve psychometric properties, since this subscale cannot be clearly assigned to dysfunctional emotion-regulation strategies (the functional value depends very much on the content of ruminative cognition) [68]. Consequently, this subscale was not utilized for analysis in this study. According to a validation study of the German form, in a clinical sample Cronbach’s α ranges between .70 and .84 in the subscales [69].

*Neuroticism* was recorded using the respective scale of the Big Five Inventory (German short form, BFI-K) [70]. The BFI-K is a questionnaire for the brief assessment of the Big Five personality factors, of which we used only the 4 items measuring *neuroticism*. Responses are scored on a 5-point scale. The retest reliability for this subscale is *r*_tt_ = .84; the estimated Cronbach’s α for the shortened form of the questionnaire is α = .67 [70].

#### Secondary outcomes

*Psychological distress* was assessed using the Hospital Anxiety and Depression Scale (German form, HADS-D) [71], an instrument for screening anxiety and depression in primarily somatically ill patients. Each of the two scales, anxiety and depression, comprises 7 items, with a 4-point scale to score responses. Reported internal consistency is α = .80 [71]. Several studies have cast doubt on the 2-factorial structure of the HADS and recommend using the overall score of the scale as a measure of psychological distress instead of anxiety and depression [72, 73]; this study therefore used psychological distress scores.

*Quality of life* was assessed via 3 visual analogue scales pertaining to physical, psychological and general well-being (0, very bad– 100, very well). These single-item scales were also used in a prior study [58]. Since they showed an excellent internal consistency (α = .96), they were combined into one scale of quality of life in the current study.

*IBS severity* was assessed with a German translation of Irritable Bowel Syndrome—Severity Scoring System (IBS-SSS), a questionnaire for clinical assessment of IBS symptom burden and severity, with higher values representing higher symptom burden. This is recommended by the Rome Foundation consensus commission [74] for the assessment of the severity and treatment effects in irritable bowel syndrome. The scale was translated by our group for this study. For this purpose, it underwent translation into German, independent back-translation into English, and comparison of both English versions for equality by an English native speaker. A translation into German and validation by another group was published later [75] and proved to be equivalent. Sound psychometric properties with regard to sensitivity, specificity and reproducibility were reported [75].

#### Demographic data and additional information

Disease duration (years since onset of IBS) and demographic data such as age, formal education, and employment status were collected in this study. Patients were also asked to report external psychological and/or psychiatric treatment, and medication. However, due to restrictions of sample size, we could not control for these factors in the analysis.

### Data analysis

**Fig 2:**
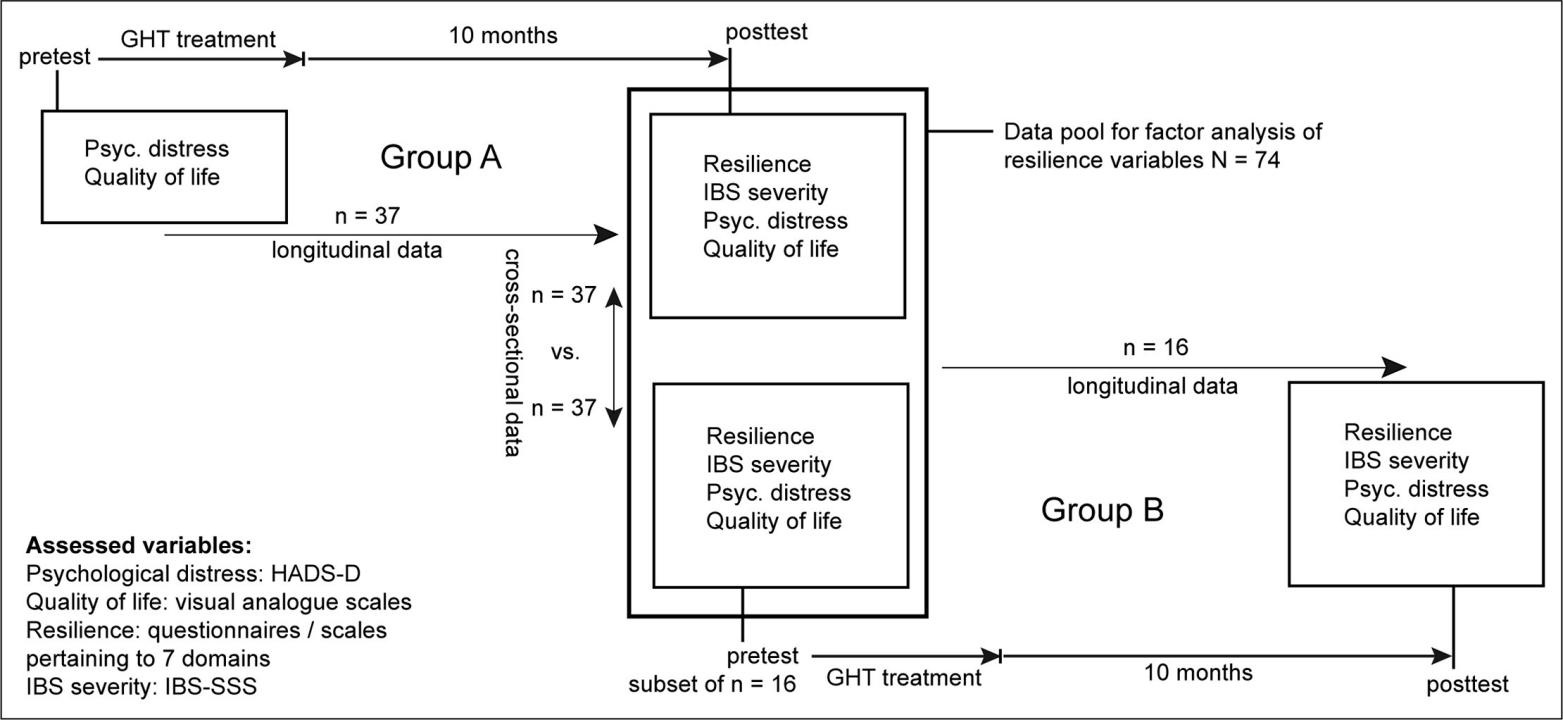
Analyses with longitudinal, cross-sectional and pooled data.

Data analysis was performed according to the per-protocol principle using IBM SPSS 22. Statistical procedures included principal component analysis for the construct validation of resilience and to obtain coefficients for the computation of a composite score for resilience. Furthermore, independent and paired *t* tests, Mann-Whitney *U* tests, and chi-squared tests were performed. Two-tailed *p* values and measures of effect size (Cohen *d*) are reported. Not all patients provided complete questionnaires. For single missing values, data were imputed by using the mean score of all item values available for that scale. If more than one item was missing per scale, no imputation was performed and no scale score was computed for that individual. The maximum of number of missing scale scores per patient was 2. Approximately 5% of final outcome values were thus missing, which is reflected in variations in the degrees of freedom of the respective statistical tests. Cross-sectional comparisons were performed with data of *n* = 37 GHT treated (Group A) and *n* = 37 untreated patients (Group B before treatment). These data were pooled for dimensional reduction of resilience domains (Fig 2). Resilience domains and IBS severity data from Group A were assessed post GHT; psychological distress and quality of life were assessed pre and post GHT. Additionally, pre and post GHT data from *n* = 16 patients in Group B who had also received GHT later on were analysed in longitudinal comparisons. In total, *N* = 74 patients were examined, of which 53 (= 37 + 16) had undergone GHT.

## Results

### Sample characteristics

Final statistical analyses were performed with data from *n* = 37 treated (Group A) and *n* = 37 untreated patients (Group B), and with *n* = 16 follow-up data from treated patients (from Group B). Overall, data of *N* = 74 individuals was used. The final total sample consisted of 54 (73%) female and 20 male individuals, with a mean age (*M* ± standard deviation [*SD*]) of 45 ± 18 years and a mean disease duration of 14 ± 13 years. In 55 cases (74%), there was a current or historic diagnosis of a psychological disorder (mostly affective and anxiety disorders); 27 (36%) made use of further psychological treatments outside the hospital, and 19 (26%) reported taking antidepressants or anxiolytics.

Baseline patient characteristics of all subgroups that had entered analyses (Group A, Group B, the treated subgroup from Group B) and of 30 patients who did not return follow-up questionnaires are provided in Table 3.

**Table 3:** Baseline characteristics of patient subgroups.

	Group A, *n* = 37	Group B, *n* = 37	Subgroup from B who obtained GHT, *n* = 16	Invited non-participants, *n* = 30
Age in years	43 (28–67)	45 (29–57)	52 (41–69)	44 (31–58)
Sex male/female	29/8	25/12	14/2	21/9
IBS disease duration in years	8 (5–18)	10 (4–20)	11 (9–28)	7 (4–10)
Presence of psychological disorder	26 (70%)	29 (78%)	12 (75%)	13 (43%)*
External psychological treatment	14 (38%)	13 (35%)	7 (44%)	15 (50%)
Antidepressant intake	10 (27%)	9 (24%)	5 (31%)	9 (30%)
Psychological distress	17 (8–23)	17 (10–24)	15 (10–22)	14 (10–20)
Quality of life	129 (62–160)	123 (67–140)	123 (74–146)	134 (77–166)

*Note*. Medians (and interquartile range) are shown for age and disease duration, psychological distress and quality of life. Otherwise, numbers are counts, with percentages in brackets. Psychological distress is the score on the Hospital Anxiety and Depression scale; quality of life was measured by visual analogue scales, with higher values indicating higher quality of life.

*p < .05, chi-squared test.

There were no differences in baseline characteristics between any of the subgroups, except a significantly lower count of known psychological diagnoses in invited non-participants (Mann-Whitney *U* tests and chi-squared tests).

### Factor analysis

Principal component analysis (PCA) was conducted on the 7 resilience domains of the pooled cross-sectional data of the *n* = 37 (treated patients; Group A) and *n* = 37 (patients before treatment; Group B) IBS patients (total *N* = 74). The Kaiser-Meyer-Olkin measure indicated that the data were adequate for PCA, KMO = .825. Bartlett’s test of sphericity, χ^2^(21) = 272.92, *p* < .001, indicated that correlations between items were sufficiently large and significant. The analysis yielded a unifactorial solution, with only one factor (‘resilience’) having an eigenvalue above 1 (i.e., 3.98), explaining 56.88% of the variance. This result was buttressed by the results of the parallel analysis, which compares the eigenvalues of the observed correlation matrix to the eigenvalues of random correlation matrices with equal numbers of observations and variables. Parallel analysis is one of the most reliable methods to determine the number of factors in PCA [76]. The 95th percentile of the first and second eigenvalues of 1000 random correlation matrices was 1.64 and 1.37, which compares to the first and second eigenvalues of the observed correlation matrix, 3.98 and 0.99. The second eigenvalue of the observed correlation matrix was markedly lower than that at the 95th percentile of random correlation matrices, which strongly suggests a unifactorial solution.

Table 4 shows the factor loadings and regression coefficients obtained from the analysis, which were subsequently used for the calculation of a weighted resilience score (see below). Core-resilience as measured by the 10 item CD-RISC displayed the highest factor loading.

**Table 4:** Resilience domains, PCA factor loadings and regression coefficients.

Resilience domain	Scale	Factor loading	Regression coefficient
Core-resilience	10-item CD-RISC	.904	.203
Self-efficacy	SWE	.851	.214
Humour	STCI scale *trait cheerfulness*	.809	.203
Neuroticism	BFI-K scale *neuroticism*	-.801	-.201
Adaptive emotion regulation	CERQ scales *acceptance*, *positive refocussing*, *planning*, *putting into perspective*, *reappraisal*	.728	.183
Dysfunctional emotion regulation	CERQ scales *self-blame*, *catastrophize*, and *other-blame*	-.604	-.152
Social support	FSozU	.498	.125

### Composite measure of resilience

Two composite measures were calculated in order to obtain an integrative measure of the different resilience domains. Eq 1 presents a naïve, unweighted composite, that is, positive appraisal domains were summed with a plus sign and absence domains with a minus sign (i.e., subtracted; this approach is also consistent with the signs for loadings in Table 4). Eq 2 presents a weighted composite, based on the regression coefficients outputted by PCA (Table 4). Applied to standardized variables, Eq 2 leads to the computation of component scores (i.e., scores that are similar to factor scores in factor analysis) which are weighted sums that are scaled to a mean of 0 and a variance of 1. In the present case, standardization of variables could not be performed, as this would have forestalled longitudinal analysis (pre and post scores would by definition each have had a mean of 0). Instead, Eq 2 was applied to the original variables in order to allow also for longitudinal analysis.

$$Unweighted\;resilience=SWE+CDRISC+STCI-BFI_{neuroticism}+CERQ_{functional}-CERQ_{dysfunctional}+FSozU$$ (1)

$$Weighted\;resilience=SWE\times .214+CDRISC\times .203+STCI\times .203-BFI_{neuroticism}\times .201+CERQ_{functional}\times .183-CERQ_{dysfunctional}\times .152+FSozU\times .125$$ (2)

### Cross-sectional comparisons

Treated patients reported significantly lower IBS severity and psychological distress, and significantly higher quality of life compared to untreated controls. There was a trend to higher values in resilience in the patients treated (Table 5).

**Table 5:** Cross-sectional comparisons between treated patients (Group A) and untreated patients (Group B).

	Group A (post treatment; *n* = 37)	Group B (untreated; *n* = 37)				
	*M* ± *SD*	*M* ± *SD*	*t*	*df*	*p*	Cohen *d*
**Positive appraisal domains**						
Core-resilience	2.23 ± 0.77	1.97 ± 0.76	1.47	70	.147	0.34
Self-efficacy	2.77 ± 0.59	2.61 ± 0.57	1.13	71	.263	0.26
Humor	2.81 ± 0.64	2.62 ± 0.51	1.42	72	.161	0.33
Adaptive emotion regulation	3.22 ± 0.81	3.05 ± 0.81	0.88	71	.382	0.20
Social support	4.00 ± 1.11	4.06 ± 0.81	0.27	72	.792	-0.06
**Absence resilience domains**						
Dysfunctional emotion regulation	2.11 ± 0.65	2.40 ± 0.59	1.98	71	.051	-0.46
Neuroticism	3.27 ± 0.94	3.69 ± 0.87	1.99	72	.051	-0.46
**Resilience composites**						
Unweighted resilience	9.71 ± 4.31	8.23 ± 3.34	1.63	70	.107	0.38
Weighted resilience	1.74 ± 0.78	1.45 ± 0.63	1.72	70	.090	0.40
**Secondary outcomes**						
Psychological distress	13.27 ± 7.85	18.71 ± 8.20	2.88	71	.045*	-0.67
Quality of life	179.36 ± 78.56	114.72 ± 61.12	3.89	68	.024*	0.90
IBS severity	196.67 ± 117.38	304.19 ± 79.57	4.55	70	< .001**	-1.06

*Note*. Values given are means and standard deviations, *t* values, degrees of freedom, and *p* values pertain to independent *t* tests. Positive values of Cohen *d* indicate higher scores in Group A, compared to Group B. Resilience composite scores were calculated as stated in Eqs 1 and 2. Psychological distress is the score on the Hospital Anxiety and Depression scale; quality of life was measured by visual analogue scales, with higher values indicating higher quality of life; IBS severity is the score on the Irritable Bowel Syndrome Severity Scoring System, with higher values indicating higher symptom burden.

*p < .05

**p < .001.

### Longitudinal comparisons

Longitudinal data of resilience and IBS severity was available for 16 patients in Group B. Resilience improved significantly (resilience composites, self-efficacy and reduced neuroticism). A parallel decrease in psychological distress, symptom burden and improvement in quality of life was observed. Longitudinal data (incomplete) was also available for psychological distress and quality of life for patients in Group A, with significant decreases in distress and improvements in quality of life (Table 6).

**Table 6:** Longitudinal comparisons pre-post GHT.

	Pre	Post				
	*M* ± *SD*	*M* ± *SD*	*t*	*df*	*p*	Cohen *d*
**Positive appraisal domains, Group B (*n* = 16)**						
Core-resilience	2.03 ± 0.79	2.14 ± 0.79	0.78	15	.450	0.20
Self-efficacy	2.64 ± 0.63	2.85 ± 0.55	2.43	15	.028*	0.61
Humor	2.57 ± 0.57	2.76 ± 0.58	1.80	15	.091	0.45
Adaptive emotion regulation	2.96 ± 0.89	3.01 ± 0.98	0.35	15	.733	0.09
Social support	4.03 ± 0.82	4.03 ± 0.80	0.02	15	.981	0.01
**Absence resilience domains, Group B (*n* = 16)**						
Dysfunctional emotion regulation	2.26 ± 0.69	2.17 ± 0.63	0.73	15	.476	-0.18
Neuroticism	3.63 ± 0.76	3.17 ± 1.04	2.70	15	.017*	-0.68
**Resilience composites, Group B (*n* = 16)**						
Unweighted resilience	8.35 ± 3.74	9.44 ± 4.00	2.34	15	.033*	0.59
Weighted resilience	1.47 ± 0.70	1.69 ± 0.75	2.61	15	.020*	0.65
**Secondary outcomes, Group B (*n* = 16)**						
Psychological distress	19.44 ± 7.46	15.69 ± 7.99	2.43	15	.028*	-0.61
Quality of life	131 ± 65	180 ± 67	2.22	13	.045*	0.59
IBS severity	302 ± 74	232 ± 96	2.35	15	.033*	-0.59
**Secondary outcomes, Group A (*n* = 37)**						
Psychological distress	16.32 ± 6.60	12.52 ± 6.77	3.63	27	.001*	-0.69
Quality of life	115 ± 55	173 ± 74	4.56	30	< .001**	0.82
IBS severity	Not available	197 ± 117	-	-	-	

Note. Values given are means and standard deviations, *t* values, degrees of freedom, and *p* values pertain to paired *t* tests. Positive values of Cohen *d* indicate higher scores post-GHT, compared to pre-GHT. Resilience composite scores were calculated as stated in Eqs 1 and 2. Psychological distress is the score on the Hospital Anxiety and Depression scale; quality of life was measured by visual analogue scales, with higher values indicating higher quality of life; IBS severity is the score on the Irritable Bowel Syndrome Severity Scoring System, with higher values indicating higher symptom burden.

*p < .05

**p < .001.

## Discussion

This study examined the structure of resilience variables in a sample of individuals suffering from IBS. Seven different factors of resilience, along with other clinical variables (psychological distress, symptom burden, quality of life), were assessed and used to validate the resilience construct. Based on the data structure observed, a composite measure of 7 resilience factors was utilized. This composite score of resilience was used to evaluate the effects of gut-directed hypnotherapy, a specific mind-body intervention for irritable bowel syndrome. The two main findings of this study were i) the unidimensional structure of resilience and ii) preliminary evidence of an improvement in resilience after GHT.

### The structure of resilience

A central problem of resilience research is the juxtaposition and lack of integration of different resilience factors. In this study, we assessed the structure of 7 important resilience factors in a routine clinical setting and a mind-body intervention administered within this setting. It is noteworthy that these resilience factors turned out to share one common latent dimension. In our opinion, this does not signify that domains of resilience are redundant; on the contrary, it highlights that resilience relates to a class of interconnected variables, with an essential range of influence for mental health and well-being.

Mental health and well-being are constituted not only by the presence of resources and positive behaviours, but also by the absence of detrimental and dysfunctional behaviours. It seems useful to consider both sides of the coin, pathology and positive psychology. With the inclusion of 2 variables from a psychopathological background, rather than from a classical resilience approach, we expanded the concept of resilience with variables that exert a salutogenetic influence by their absence. We added *dysfunctional emotion regulation* and *neuroticism* to the positive resilience variables *conviction to adapt and overcome hardship*, *self-efficacy*, *humour/positive emotionality*, *adaptive emotion regulation* and *perceived social support* in our measurement approach. To some extent this is a departure from the salutogenetic perspective, but the recognition of suppression or absence of negative appraisals and emotion is in line with recent conceptual developments in resilience research [22].

Furthermore, the results of PCA in this study provide support for the 10 item-version of the Connor-Davidson Resilience Scale; the high loading on the overall dimension of resilience confirmed that this scale measures important aspects of resilience.

### The effects of gut-directed hypnotherapy

As was hypothesized, IBS patients showed an improvement in resilience after gut-directed hypnotherapy. The strongest effects were observed for the composite score of resilience. This was to be expected, as the reliability of an aggregated measure increases with the number of individual measurements. The strongest effects in individual domains were observed with regard to self-efficacy (increased), and neuroticism (decreased). In parallel, improved quality of life and decreased psychological distress and IBS severity were observed. This pattern was significant in the longitudinal data. There were similar trends in cross-sectional comparisons between treated and untreated IBS patients.

As a result of the broad class of behaviours and variables affected, an improvement in resilience can make a substantial difference to the individual’s situation. A number of seminal experiments have shown the significance of resilience variables as self-efficacy appraisals for the ability to withstand and cope with aversive situations, and even for pain perception [77, 78]. The marked reductions in symptom severity and psychological distress and improvements in well-being that were observed synchronously with the improvement in resilience in this study strongly support an improvement in successful coping. The improvement of resilience thus seems a likely pathway of action of gut-directed hypnotherapy. However, the observations made in this study are merely correlational and causation by hypnotherapy cannot be concluded. Clearly, direct data from larger samples on the mediating effects of increases of resilience by GHT on the reduction of IBS-symptoms are needed.

### Limitations and strengths of the study

Limitations of this study are to be seen in the high proportion of missing data and the low rate of response to the survey, especially in patients who had been contacted via mail and/or telephone. However, baseline analyses provided some assurance against biased characteristics of non-participants. Recruitment stopped before previously calculated sample sizes required for adequate statistical power were achieved, and due to the study design and setting, only a small number of subjects provided full longitudinal data. The small sample size and lack of power must therefore be considered a limitation of this study. Constraints also arise from the relatively large number of patients undergoing external psychotherapy in addition to the study intervention and the intake of antidepressants during the study. The factor analysis of resilience was based on a dataset 50% pre- and 50% post-hypnotherapy patients, and the sample size was at the lower limit for this kind of analysis. Independent replications of our results with larger sample sizes are therefore needed.

Other important factors of resilience, such as optimism [3], sense of purpose [16], behavioural coping [5] and mindfulness [18] were not taken into account in this study. Nor could disease duration, external psychological and/or psychiatric treatment and medication or demographic variables such as age, formal education and employment status be controlled for in the analysis. Since there were no procedures such as randomization of study subjects, or blinding of investigators, the study does not fulfill advanced methodological quality criteria.

On the other hand, strengths of the study are its high degree of ecological validity due to a real-world setting in tertiary care, and the examination of a typical sample of IBS patients with a high psychological burden. The study contributed to the integration and validation of resilience, and it illustrates a method of empirically integrating resilience factors by dimensional reduction. Furthermore, it demonstrates the efficacy of gut-directed hypnotherapy, a relatively simple and cost-effective intervention in the highly distressed and health-care-using population of IBS patients. Making use of the composite resilience measure, it makes a contribution to the issue of pathways of action in this therapy. To our knowledge, after Lindfors et al.’s study with negative results, this is the first study yielding evidence of a resilience-enhancing effect of GHT.

## Conclusion

The empirically examined resilience domains turned out to be unidimensional, thus all shared a latent dimension of resilience. This reflects the close functional relatedness of resilience domains and emphasizes the significance of this class of variables for well-being and health, as a potential treatment goal. The inclusion of ‘absence’ variables from a psychopathological background, contributing to health and well-being by negative manifestation, seems reasonable. Further evaluation of interventions aimed at strengthening psychological resources and resilience is required, as well as an ongoing integrational effort in the field of resilience.

An improvement in resilience occurred in the course of gut-directed hypnotherapy. Further studies are required to confirm the potential role of increased resilience as a pathway of action of hypnotherapy.

## Supporting information

S1 Appendix ClinicalTrials.gov Registration. (PDF)

S2 Appendix Study protocol (German). (PDF)

S3 Appendix Study protocol (English translation). (PDF)

S4 Appendix TREND Checklist. (PDF)
