# Supplementary material for: The structure of resilience in irritable bowel syndrome and its improvement through hypnotherapy: Cross-sectional and prospective longitudinal data
Source: PLoS One. 2018 Nov 12;13(11):e0202538. doi: 10.1371/journal.pone.0202538 (PMC6231615; doi:10.1371/journal.pone.0202538)
Supplement: S1 Appendix — (PDF) [file pone.0202538.s001.pdf]

ClinicalTrials.gov Protocol Registration and Results System (PRS) Receipt
Release Date: 04/12/2016

ClinicalTrials.gov ID: NCT02737410

Study Identification

Unique Protocol ID: 1488/2012

Brief Title: Resilience in Irritable Bowel Syndrome and Gut-focused Hypnotherapy

Official Title: Resilience in Irritable Bowel Syndrome and Gut-focused Hypnotherapy: Longitudinal Study With Hypnotherapy Patients and Cross Sectional Control Group

Secondary IDs:

Study Status

Record Verification: April 2016

Overall Status: Completed

Study Start: June 2012

Primary Completion: June 2014 [Actual]

Study Completion: October 2014 [Actual]

Sponsor/Collaborators

Sponsor: Medical University of Vienna

Responsible Party: Principal Investigator
Investigator: Gabriele Moser [gmoser]
Official Title: Professor
Affiliation: Medical University of Vienna

Collaborators:

Oversight

FDA Regulated?: No

IND/IDE Protocol?: No

Review Board: Approval Status: Approved
Approval Number: 1488/2012
Board Name: Ethikkommission der Medizinischen Universität Wien
Board Affiliation: Medizinische Universität Wien
Phone: 0043 1 40400 21470
Email: ethik-kom@meduniwien.ac.at

Data Monitoring?: No

Plan to Share Data?: Yes
via www.figshare.com

Oversight Authorities: Austria: Ethikkommission

Study Description

Brief Summary: Background:

Resilience refers to a class of variables highly relevant for wellbeing and coping with stress, trauma, and chronic adversity. Despite its significance for health, resilience is hardly examined empirically and suffers from poor conceptual integration. Irritable bowel syndrome (IBS) is a functional disorder with altered psychological stress reactivity and brain-gut-microbiota axis, which causes high chronic strain. Gut-focused hypnotherapy (GHT) is a standardized treatment for IBS targeting at resilience. An increase of resilience by GHT has been hypothesized but requires further investigation.

Aims of the study were construct validation and development of an integrational measure of different resilience domains by dimensional reduction, and investigation of change in resilience in IBS patients by GHT.

N=74 Gastroenterology outpatients with Irritable Bowel Syndrome (Rome III criteria) were examined in 7 resilience domains, quality of life, psychological distress and symptom severity. n=53 of these participate in 7 to 10 Gut-directed Hypnotherapy group sessions (Manchester protocol). Post-treatment examinations were performed 10 months after last GHT session.

Detailed Description: Aims of this study were, to measure detailed factors of resilience by adequate psychological instruments in a sample of Irritable Bowel Syndrome (IBS) patients and to investigate whether they are facets of the same underlying construct. To calculate a composite measure of resilience based on obtained insights concerning the structure of resilience. To test the hypothesis of a positive relation between resilience and response to therapy. And finally, to investigate differences between patients untreated or treated with Gut-directed Hypnotherapy in groups cross-sectionally and longitudinally, to test the hypothesis of a presumed increase in resilience (using the composite score) with changes in IBS symptoms, quality of life and psychological distress in parallel.

Study location and recruitment:

N=74 Irritable bowel syndrome patients (diagnosed according to Rome III criteria) aged between 18 and 70 and refractory to other therapies were recruited at the Specialized outpatient-clinic for Psychosomatics at the Division of Gastroenterology and Hepatology, Department for Internal Medicine III, University Hospital of Vienna. Antidepressants or anxiolytics and/or ongoing psychotherapy were allowed since severe comorbid psychological problems are a common problem in IBS patients. The study protocol was approved by the ethics committee of the Medical University of Vienna (ID: 1488/2012). Informed consent in writing was given by each participant.

Study Design and Treatment:

Cross-sectional comparisons were performed with data of n = 37 GHT treated and n = 37 untreated patients (control group) post GHT. This data were pooled for dimensional reduction of resilience domains. Resilience and IBS severity data from the treatment group were assessed post GHT; psychological distress and quality of life were assessed pre and post GHT. A fraction of n = 16 of the untreated patients (former control group) subsequently also received GHT treatment. Pre and post GHT data of these patients were collected and then used for longitudinal comparisons, along with the pre and post GHT data on psychological distress and quality of life of the primary treatment group. In total, N= 74 patients were examined, in total 53 (= 37 + 16) of which underwent GHT. Post treatment examinations were performed 10 months after last GHT session. The GHT protocol used was the Manchester protocol of GHT and consisted of 10 weekly sessions (45 min), with six patients per group over a treatment period of 12 weeks. GHT was performed at the University Hospital by two experienced physicians trained in Manchester (UK).

Conditions

Conditions: Irritable Bowel Syndrome

Keywords: Resilience
Coping
Psychosomatic
Hypnosis
Construct Validation

Study Design

Study Type: Interventional

Primary Purpose: Basic Science

Study Phase: N/A

Intervention Model: Parallel Assignment

Number of Arms: 2

Masking: Open Label

Allocation: Non-Randomized

Endpoint Classification: N/A

Enrollment: 74 [Actual]

Arms and Interventions

Arms	Assigned Interventions
Active Comparator: Treatment<br>Treatment group obtaining Gut-directed Hypnotherapy	Behavioral: Gut-directed Hypnotherapy<br>The GHT protocol used was the Manchester protocol of GHT and consisted of 10 weekly sessions (45 min), with six patients per group over a treatment period of 12 weeks. GHT was performed at the University Hospital by two experienced physicians trained in Manchester (UK).<br>Other Names:<br>• Gut-focused Hypnotherapy
No Intervention: Control<br>Control group	

Outcome Measures

Primary Outcome Measure:

1. Resilience assessed using the Connor-Davidson Resilience Scale
   [Time Frame: 1 year] [Safety Issue: No]
   10-item Connor-Davidson Resilience Scale, with higher values indicating higher resilience.

2. Self-efficacy assessed by the Skala zur allgemeinen Selbstwirksamkeitserwartung
   [Time Frame: 1 year] [Safety Issue: No]
   Skala zur allgemeinen Selbstwirksamkeitserwartung (SWE) is a german questionnaire to assess self-efficacy. Higher values mean higher self-efficacy.

3. Humor assessed by the State-Trait Cheerfulness Inventory
   [Time Frame: 1 year] [Safety Issue: No]

State-Trait Cheerfulness Inventory, german version (STCI); higher values indicate higher predisposition to experience positive emotion by humor.

4. Social support assessed by the Fragebogen zur Sozialen Unterstützung
   [Time Frame: 1 year] [Safety Issue: No]
   Fragebogen zur Sozialen Unterstützung (F-SozU), a german Questionnaire assessing perceived social support, with higher values indicating higher perceived support.

5. Emotion regulation assessed by the Cognitive Emotion Regulation Questionnaire
   [Time Frame: 1 year] [Safety Issue: No]
   Cognitive Emotion Regulation Questionnaire (CERQ), german version; assessing adaptive and dysfunctional cognitive reactions to aversive events. Higher values represent higher occurence of respective behaviours.

6. Neuroticism assessed by the Big Five Inventory
   [Time Frame: 1 year] [Safety Issue: No]
   Big Five Inventory, german short form (BFI-K), for assessment of neuroticism. Higher values indicate higher neuroticism.

Secondary Outcome Measure:

7. Psychological distress assessed by the Hospital Anxiety and Depression Scale
   [Time Frame: 1 year] [Safety Issue: No]
   The Hospital Anxiety and Depression Scale (German version, HADS-D) is an instrument for screening anxiety and depression in primarily somatic ill patients. Higher values indicate more distress.

8. Quality of life assessed by visual analogue scales
   [Time Frame: 1 year] [Safety Issue: No]
   Quality of life was assessed via single visual analogue scales (VAS). Higher values represent higher wellbeing.

9. Symptom severity assessed by the Irritable Bowel Syndrome - Severity Scoring System
   [Time Frame: 1 year] [Safety Issue: No]
   The Irritable Bowel Syndrome - Severity Scoring System (IBS-SSS) is a questionnaire for clinical assessment of IBS symptom burden and severity. Higher values indicate higher symptom burden.

Eligibility

Minimum Age: 18 Years

Maximum Age: 70 Years

Gender: Both

Accepts Healthy Volunteers?: No

Criteria: Inclusion Criteria:

- a diagnosis of IBS (Rome-III-criteria)
- Indication of GHT because of no adequate relief of IBS symptoms and no improvement of disease-related quality of life through other IBS therapies

Exclusion Criteria:

- pregnancy, mental retardation
- insufficient knowledge of German
- transit time from home to hospital longer than one hour

Contacts/Locations

Study Officials: Gabriele Moser, Professor
Study Principal Investigator
Medical University of Vienna

Locations: Austria
Medical University of Vienna
Vienna, Austria, 1090

References

Citations:

Links:

Study Data/Documents:

U.S. National Library of Medicine | U.S. National Institutes of Health | U.S. Department of Health & Human Services
