# Supplementary material for: The structure of resilience in irritable bowel syndrome and its improvement through hypnotherapy: Cross-sectional and prospective longitudinal data
Source: PLoS One. 2018 Nov 12;13(11):e0202538. doi: 10.1371/journal.pone.0202538 (PMC6231615; doi:10.1371/journal.pone.0202538)
Supplement: S2 Appendix — (PDF) [file pone.0202538.s002.pdf]

Protokoll Resilienz RDS GHT Version 2.4 vom 6.8.2012

25.7.2012

Resilienzfaktoren bei Reizdarmsyndrom und gut-focused Hypnosetherapie. Längsschnittuntersuchung an Patienten in Hypnosetherapie mit Querschnittskontrollgruppe.

Dr. Ulrich Tran, Universität Wien,
Fakultät für Psychologie

Johannes Peter

Prof. Dr. Gabriele Moser,
Medizinische Universität Wien

Abstract.

Hintergrund: Psychologische Faktoren spielen bei Reizdarmsyndrom eine bedeutende Rolle in Krankheitswahrnehmung und Schmerzmodulation. Es wird angenommen, dass kognitive und Persönlichkeitsmerkmale als Resilienzfaktoren sowohl maladaptiven als auch protektiven Einfluss hinsichtlich zentraler Größen des Krankheitsgeschehens haben können. Die gut-focused Hypnosetherapie gilt als wirksame Therapiemethode bei Reizdarmsyndrom (RDS). Fraglich ist, inwieweit sich Faktoren der Resilienz auf den Therapieerfolg auswirken und wie die GHT auf Größen der Resilienz rückwirkt.

Ziele der Studie: Erkenntnisgewinn hinsichtlich typischer psychologischer Charakteristika bei RDS. Ermittlung kognitiver und persönlichkeitsbezogener Faktoren der Resilienz im Wirkgefüge des RDS und deren therapeutische Veränderung.

Methodik: Eine Erhebung an n_{ges}= 80 Patienten hinsichtlich folgender Variablen: LQ, wahrgenommene Schwere der Symptomatik, Angst und Depression und Maße der Resilienz. Die Erhebung teilt sich in zwei Gruppen auf: n_A= 40 Patienten erhielten routinemäßig 10 Sitzungen GHT, welche von von bislang 3 längsschnittlichen Erhebungen in Variablen der Lebensqualität und der affektiven Belastung flankiert wurde. In einem katamnestischen follow-up sollen nun zusätzlich Ausprägungen in Resilienzvariablen gemessen werden. Ergänzend soll an n_B= 40 Patienten, welche an der gut-focused Hypnose teilnehmen werden, eine Querschnittserhebung der fraglichen Variablen durchgeführt werden.

Inhaltsverzeichnis

Abstract. 2
Projektmitarbeiter 3
- Dr. Ulrich Tran 3
- Johannes Peter 3
- Univ.-Prof. Dr. Gabriele Moser 3

Autorenschaft 3
Wissenschaftlicher Hintergrund 3
- Ziele und Hypothesen der Studie 4
- Studiendesign 5
- Stichprobe 5
- Intervention 7
- Ergebnisvariablen 7

Statistik und Datenanalyse 8
- Stichprobengröße 8

Ethische Gesichtspunkte 8
Literaturverzeichnis 9
Anhang 10

Projektmitarbeiter

Dr. Ulrich Tran
Institut für Psychologische Grundlagenforschung und Forschungsmethoden
Liebiggasse 5
1010 Wien
ulrich.tran@univie.ac.at

Johannes Peter
Student der Psychologie, Universität Wien
a0609894@unet.univie.ac.at

Univ.-Prof. Dr. Gabriele Moser
Universitätsklinik für Innere Medizin III
Abteilung für Gastroenterologie und Hepatologie
Währinger Gürtel 18-20
1090 Wien
gabriele.moser@meduniwien.ac.at

Autorenschaft

Erstautor, Corresponding Author	Dr. Ulrich Tran
Zweitautor	Johannes Peter
Seniorautor	Prof. Dr. Gabriele Moser

Wissenschaftlicher Hintergrund

Reizdarmsyndrom (RDS) ist gekennzeichnet durch unangenehme Empfindungen, viszeralen Schmerz und gestörte Defäkation ohne bekannten körperlichen Grund. Diese Symptomatik wird vermutlich durch Dysregulation der ‚brain-gut axis' auf verschiedenen Ebenen verursacht. Neben immunologischen Faktoren und Veränderungen im Bereich des autonomen Nervensystems spielen zentralnervöse und psychologische Prozesse eine bedeutende Rolle.

Psychologische Aspekte bei RDS umfassen nicht nur hohe Levels von Distress, pychischen Komorbiditäten und erhöhte Angst- und Depressionspegel, sondern auch auf körperliche Variablen rückwirkende Verarbeitungsmechanismen. Im Lichte einer kognitiven Neurobiologie von RDS (Kennedy et al., 2012) sind kognitive und persönlichkeitsbezogene Faktoren entscheidend hinsichtlich der Modulation von Schmerz und der Wahrnehmung zentraler krankheitsbezogener Outcomes wie gesundheitsbezogener Lebensqualität und psychologischem Distress. Zu den wichtigsten Faktoren zählen neben dysfunktionalen

Kognitionen und subjektiven Krankheitstheorien Aspekte der Persönlichkeit. Letztere beziehen sich gleichermaßen auf problematische Verarbeitungsstile wie z.B. Somatisierungstendenzen als auch auf protektiv wirksame Resilienzfaktoren.
Gut-directed Hypnosetherapie (GHT) hat sich in zahlreichen Untersuchungen als nachhaltig wirksame Therapiemethode bei RDS erwiesen (vgl. z.B. Lindfors et al., 2012; Webb, Kukuruzovic, Catto-Smith, & Sawyer, 2007; Whorwell, Prior & Faragher, 1984). Die GHT beinhaltet die Induktion von tiefer Entspannung und Trance, sowie Suggestionen und Imaginationen, die therapeutische Veränderungen auf der Ebene von Persönlichkeit, kognitiven Verarbeitungsmustern und krankheitsbezogenen Attributionen bewirken sollen.
Somit eröffnet sich ein breites Spektrum möglicher Wirkmechanismen der GHT, von kognitiven und emotionalen Veränderungen bis hin zu verbesserter Modulation auf der Ebene des autonomen Nervensystems. Zahlreiche Wirksamkeitsstudien dokumentieren neben Lebensqualitätverbesserungen und verringertem Schmerzerleben Rückgänge in affektiven Belastungsvariablen wie Angst und Depression. Neben unmittelbaren zerebralen Verbesserungen der Schmerzverarbeitung (Faymonville et al., 2003) spielen hinsichtlich der Wirksamkeit der GHT vermutlich kognitive Mechanismen eine fundamentale Rolle. Vereinzelt konnten Veränderungen in kognitiven Variablen durch die gut-focused Hypnose bereits demonstriert werden (Gonsalkorale, Toner & Whorwell, 2004; Palsson, Turner, Johnson, Burnett & Whitehead, 2002).
Die Erforschung solcher Faktoren ist im Kontext von psychologischer Resilienz hoch relevant. Erstens stellen RDS-Patienten eine besonders risikoexponierte Gruppe dar, da ihr Leiden chronisch ist und zudem keine unmittelbare organische Ursache aufweist. Zweitens wirft die hohe Komorbidität von RDS und psychischen Erkrankungen die Frage auf, ob diese teilweise auf identen Mechanismen beruhen. Naliboff et al. (2012) konnten mittels Strukturgleichungsmodellen zeigen, dass psychologische Faktoren ursächliche Auswirkung auf die gesundheitsbezogene Lebensqualität bei RDS haben. Umgekehrt ist bekannt, dass Aspekte psychologischer Resilienz einen Beitrag zur Aufrechterhaltung somatischen Wohlbefindens leisten können (Ickovics et al., 2006).
Fortschreitende Konzeptualisierung und Identifikation spezifisch relevanter Reslienzfaktoren für bestimmte Gruppen wie hier der Population der Reizdarmpatienten sind erklärte Ziele der Resilienzforschung (Davydoff, Stewart, Ritchie & Chaudieu, 2009).
Das im Rahmen der vorliegendenden Arbeit geplante Projekt soll sich diesem Forschungsdesiderat verpflichten und einen Beitrag zur Konzeptualisierung von Resilienz, dem Verständnis des RDS und den Mechanismen der GHT leisten.

Ziele der Studie

Durch die Messung von Veränderungen in Schweregrad des RDS, Lebensqualitäts- und affektiven Variablen soll die Wirksamkeit der GHT dokumentiert werden. Hinsichtlich der erhobenen Resilienz- und Persönlichkeitsvariablen liegen bislang keine Untersuchungen an der Population der RDS-Patienten vor. Die Untersuchung soll aufklären, welchen Faktoren besondere Bedeutung für das Krankheitserleben und mögliche therapeutische Veränderungen beizumessen ist. Durch Gegenüberstellung zur Kontrollgruppe soll Rückschluss gezogen werden auf Veränderungen in Maßen der Resilienz durch die GHT.

Zentrale Ziele/Hypothesen:

1. Verminderte Belastung (Schweregrad, Affektive Variablen, LQ) infolge der GHT
2. Explorative Erhebung von Resilienzvariablen bei Reizdarmpatienten
3. Resilienz geht mit vermindertem Belastungserleben einher
4. Höhere Ausprägung von Resilienz nach GHT
5. Höhere Ausprägung von Resilienz bei starken Therapieansprechern
6. Erfolgserwartung bez. Therapie korreliert mit Resilienzfaktoren
7. Erfolgserwartung korreliert mit Therapieerfolg

Studiendesign

Als Studiendesign ist eine Kombination aus einer längsschnittlichen Untersuchung von Patienten in Hypnosetherapie und einer Querschnittsuntersuchung an Patienten, welche auf die Hypnosetherapie warten geplant. Für die Längsschnittuntersuchung wurden routinemäßig bereits drei Erhebungen durchgeführt (t1-t3), lediglich die follow-up Untersuchung ($t4_A$) und die Erhebung an der Kontrollgruppe ($t4_B$) sind noch ausständig.

A) Längsschnittliche Erhebung von Lebensqualitätvariablen, sowie Angst und Depression und bei n_A= 40 Patienten mit Reizdarmsyndrom, welche Gruppenhypnosetherapie erhalten. Insgesamt vier Messzeitpunkte: Baseline(t1), während der Gruppenhypnosetherapie (t2), am Ende der Therapie (t3).
Katamnese ($t4_A$) mit zusätzlicher Untersuchung von ausgewählten Faktoren der Resilienz und RDS-Symptomatik.

B) Querschnittserhebung ($t4_B$) bei n_B= 40 RDS-Patienten ohne psychologische Behandlung. Erhebungsmaße analog zu $t4_A$: Lebensqualitätvariablen, Angst und Depression, Resilienzfaktoren, RDS-Symptomatik .

Zur Veranschaulichung des Designs siehe Grafik im Anhang (Abb.1).

Direkte Kausalschlüsse können mit diesem Design nicht gezogen werden. Für die epistemische Gültigkeit des Vergleichs spricht von VG und KG spricht jedoch die aus der Literatur bekannte relative Zeitstabilität von Resilienz- und Persönlichkeitsfaktoren. Um die Vergleichbarkeit der beiden Gruppen zu gewährleisten, sollen die Charakteristika von VG und KG soweit als möglich angeglichen werden.

Stichprobe

Die Untersuchung soll sich auf Daten von Reizdarmpatienten der Psychosomatischen Ambulanz der Univ. Klinik, Abteilung für Innere Medizin III stützen. Teilweise kann auf bereits vorliegende Erhebungsergebnisse zugegriffen werden. Die Einwilligung zur Datenerhebung und Auswertung wird bei der Ethikkommission der Medizinischen Universität Wien eingereicht.

1. Bereits vorliegende Datensätze
Dabei kann auf im Zeitraum von 2009 bis heute routinemäßig erhobene Daten von Reizdarmpatienten, welche Gruppenhypnose erhielten zurückgegriffen werden. Von diesen

Patienten wurden jeweils zu folgenden Zeitpunkten Daten erhoben: prospektiv vor der ersten Sitzung, zur 5. und zur 10. (letzten) Sitzung. Abgefragt wurden Selbsteinschätzungen auf visuellen Analogskalen (VAS) in den Dimensionen: körperliches Befinden, seelisches Befinden, sowie allgemeines Befinden und Lebensqualität. Zum ersten Zeitpunkt der Vorgabe wurde zudem die subjektive Erwartung von Veränderungen auf genannten Dimensionen erfragt, zu den beiden übrigen Messzeitpunkten eine Einschätzung relativ zur Ausgangslage vor Beginn der GHT, jeweils wiederum auf VAS. Zu allen drei Zeitpunkten wurde die *HADS-D* (*Hospital Anxiety and Depression scale – Deutsch*) zur Erfassung von Angst und Depression vorgegeben.
Die Fragebögen aus diesen Erhebungen liegen in den Ambulanzakten der Patienten auf. Nach Genehmigung durch die Ethikkommission sollen die Daten von n_A= 40 Patienten eingesehen, einer anonymisierten statistischen Auswertung zugeführt und von einer katamnestischen Nachuntersuchung gefolgt werden.

2. Geplantes weiteres Procedere
Mit n_A= 40 jener Patienten, von denen bereits Daten vorliegen soll eine telefonische Kontaktaufnahme erfolgen, im Rahmen derer eine kurze Erklärung des Vorhabens und die Bitte um Erlaubnis zur Zusendung von Fragebögen kommuniziert werden soll. Bei Ablehnung soll telefonisch lediglich die Frage gestellt werden, ob durch die GHT eine angemessene Besserung eingetreten ist (zu beantworten mit ja/nein). Ansonsten werden nach erfolgter mündlicher Einwilligung die Fragebögen zur Rücksendung an die Pat. versandt. Die Sendung enthält neben den Fragebögen vorfrankierte Umschläge zur Rücksendung sowie die Patienteninformation und das Formular zur Erteilung der schriftlichen Einwilligung zur Studienteilnahme (informed consent).

3. Weiters sollen n_B= 40 Patienten an der Ambulanz um Teilnahme an der Studie gebeten werden. Nach mündlicher Erläuterung des Forschungsvorhabens erhalten diese die Fragebögen direkt zum Ausfüllen an der Ambulanz. Das schriftliche Einverständnis zur Teilnahme soll bei diesen Pat. beim Ausfüllen der Fragbögen eingeholt werden.

Für beide Patientengruppen soll folgendes gelten:
Telefonnummern des Diplomanden Herrn Peter und der Ambulanz (Univ. Prof. Moser) werden für Rückfragen bekanntgegeben. Zusätzlich wird angeboten, die Fragebögen telefonisch oder nach Terminabsprache an der Ambulanz gemeinsam mit dem Diplomanden bzw. in Gegenwart von Frau Prof. Moser durchzuarbeiten. Es wird darauf hingewiesen, dass Frau Prof. Moser bei auftauchendem Bedürfnis nach einem Gespräch an der Ambulanz zur Verfügung steht oder zurückruft.

3. Ein- und Ausschlusskriterien

**Einschlusskriterien**
RDS-Patienten nach den Rom-III-Kriterien<br>Altersbereich: 18-70 Jahre
**Ausschlusskriterien**
Pat. mit akuten Komplikationen, Darmoperationen<br>Pat. mit starken Depressionen oder Angstzuständen<br>Pat. mit unzureichenden Deutschkenntnissen

Pat. in Schwangerschaft

Intervention

Im Rahmen der Studie ist zum jetzigen Zeitpunkt keine Intervention vorgesehen. Die Hälfte der Patienten der Stichprobe hat bereits routinemäßig an der bauchgerichteten Gruppenhypnosetherapie teilgenommen. Diese wurde an der Psychosomatikambulanz der Univ. Klinik für Innere Medizin III durchgeführt, jeweils 10 Sitzungen à 45 Minuten in wöchentlicher Frequenz, in Gruppen von 6 bis 8 Patienten.
Die Hypnose wurde nach den Vorgaben des *Manchester-Protokolls* der „gut-focused hypnotherapy" durchgeführt (Gonsalkorale, 2006). Diese kann folgendermaßen umschrieben werden: nach einer kurzen Erläuterung der Pathophysiologie der Erkrankung wird eine hypnotische Trance induziert. Im Rahmen dieser Trance werden Anleitungen zu tiefer Entspannung, taktiler Eigenberührung (Auflegen der Hände auf den Bauch), Imaginationen und Suggestionen gegeben. Diese zielen auf ein Entkatastrophisieren krankheitsbezogener Vorstellungen, das Wiedererlangen subjektiver Kontrolle über körperliche Zustände, die Verringerung von Angst und Stress und das Erlernen wirksamer Selbstberuhigung ab.

Ergebnisvariablen

Für die Erhebung von Lebensqualität, affektiver Belastung, Schwere der Krankheitssymptomatik und Resilienzfaktoren an Hypnose- und Kontrollgruppe sollen verschiedene Fragebogenverfahren zum Einsatz kommen.

**Lebensqualität & affektive Variablen**
• körperliches Befinden, seelisches Befinden, sowie allgemeines Befinden und Lebensqualität (VAS)<br>• Einschätzung hins. Erwartung und eingetretenem Erfolg der Hypnose (nur Hypnosegruppe)<br>• Angst und Depression (HADS-D, Zigmond & Snaith, 1983)
**Schwere der Krankheitssymptomatik**
• IBS-Symptom Severity Scale, deutsche Übersetzung<br>• Frage nach adäquater Besserung (adequate relief- dichotomes Antwortformat)
**Resilienzfaktoren**
• BFI-K Big Five Persönlichkeit, Faktoren Extraversion und Neurotizismus (Rammstedt & John, 2005)<br>• Trait Cheerfulness (Ruch, Köhler, & van Thriel, 1996)<br>• SWE Selbstwirksamkeitserwartung (Schwarzer & Jerusalem, 1999)

- CD-RISC Resilienzskala – Kurzfassung (Connor & Davidson, 2003)
- CERQ Kognitive Emotionsregulation (Loch, Hiller, & Witthöft, 2011)
- F-SozU K-14 Soziale Unterstützung (Fydrich, Sommer, Tydecks,& Brähler, 2009)

Statistik und Datenanalyse

Daten aus Fragebögen und Patientenakten werden im Zuge der computerisierten Datenverarbeitung mit Codes versehen und anonymisiert weiterverarbeitet.
Die statistische Auswertung soll mit SPSS (Version 16 oder höher) durchgeführt werden. Die Auswertung der erlangten Daten wird teils deskriptivstatistisch erfolgen, für die wichtigsten Hypothesen (z.B. Veränderung in Lebensqualität, Angst und Depression in Folge von GHT) werden ausserdem inferenzstatistische Auswertungsverfahren zur Anwendung kommen. Vorgesehen sind hierfür multiple Regressionen, Pearson Korrelationen, faktorielle Varianzanalysen und multivariate Varianzanalysen für wiederholte Messungen (repeated-measures MANOVAs) für die längsschnittliche Auswertung der Hypnosegruppe.

Stichprobengröße

In einer am AKH von Frau Prof. Moser rezent durchgeführten Untersuchung an Reizdarmpatienten unter gut-focused Gruppenhypnose (zur Publikation eingereicht) zeigten sich im Fragebogen SF-36 zur gesundheitsbezogenen Lebensqualität (McHorney, Ware & Raczek, 1993) nach 12 Monaten im Vergleich zu Patienten in Standardtherapie Effektstärken von durchschnittlich $r = .34$; mit dem Statistikprogramm G*Power 3.1 (Faul, Erdfelder, Buchner & Lang, 2009) wurde mit diesen Werten folgendes berechnet: bei einem Alpha-Fehlerniveau von 5 % und einer Power von .80 ergibt sich ein zu fordernder Stichprobenumfang von etwa 49 Personen.
Flik et al. (2011) haben in der Publikation ihres Studienprotokolls für eine valide katamnestische Gegenüberstellung von Patienten nach GHT und Placebokontrollgruppe eine Stichprobengröße von 44 Personen veranschlagt.
Die Konzeption der Studie mit 40 Personen pro Gruppe erfüllt diese Anforderungen zumindest annähernd.

Ethische Gesichtspunkte

Für die Patienten ist die Befragung mit einem gewissen Zeitaufwand für das Ausfüllen der Fragebögen verbunden. Die aufzubringende Zeit und die Bekanntgabe von persönlichen Einstellungen kann mitunter als unangenehm empfunden werden. Demgegenüber steht die positiv besetzte Möglichkeit, zum Erkenntnisfortschritt beizutragen – im Allgemeinen sowie hinsichtlich der eigenen Erkrankung.
Ein gesundheitliches Risiko besteht beim Ausfüllen der Fragebögen nicht.

Literaturverzeichnis

Connor, K. M., & Davidson, J. R. T. (2003). Development of a new resilience scale: The Connor–Davidson Resilience Scale (CDRISC). *Depression and Anxiety*, *18*, 76-82.

Davydoff, D.M., Stewart, R., Ritchie, K., & Chaudieu, I. (2010). Resilience and mental health. *Clinical Psychology Review, 30,* 479–495.

Faul, F., Erdfelder, E., Buchner, A., & Lang, A.-G. (2009). Statistical power analyses using G*Power 3.1: Tests for correlation and regression analyses. *Behavior Research Methods, 41*, 1149–1160.

Faymonville, M. E., Roediger, L., Del Fiore, G., Delgueldre, C., Phillips, C., Lamy, M., . . . Laureys, S. (2003). Increased cerebral functional connectivity underlying the antinociceptive effects of hypnosis. *Cognitive Brain Research, 17*(2), 255-262. doi: 10.1016/s0926-6410(03)00113-7

Flik, C.E., van Rood, Y.R., Laan, W., Smout, A.J., Weusten, B.L., Whorwell, P.J., & de Wit, N.J. (2011). A randomised controlled trial on hypnotherapy for irritable bowel syndrome: design and methodological challenges (the IMAGINE study). *Gastroenterology, 11*(137)*,* 1–10.

Francis, C.Y., Morris, J., & Whorwell PJ. (1997). The irritable bowel severity scoring system: a simple method of monitoring irritable bowel syndrome and its progress. *Alimentary Pharmacology & Therapeutics, 11*, 395–402. doi: 10.1046/j.1365-2036.1997.142318000.x

Fydrich, T., Sommer, G., Tydecks, S., & Brähler, E. (2009). Fragebogen zur sozialen Unterstützung (FSozU):Normierung der Kurzform (K-14). *Zeitschrift für Medizinische Psychologie*, *18*, 43-48.

Gonsalkorale, W. M. (2006). Gut-directed hypnotherapy: The Manchester approach for treatment of irritable bowel syndrome. *International Journal of Clinical and Experimental Hypnosis, 54*(1), 27-50. doi: 10.1080/00207140500323030

Gonsalkorale, W. M., Toner, B. B., & Whorwell, P. J. (2004). Cognitive change in patients undergoing hypnotherapy for irritable bowel syndrome. *Journal of Psychosomatic Research, 56*(3), 271-278. doi: 10.1016/s0022-3999(03)00076-x

Ickovics, J.R., Milan, S., Boland, R., Schoenbaum, E., Schuman, P., & Vlahov, D. (2006). Psychological resources protect health: 5-year survival and immune function among HIV-infected women from four US cities. *AIDS, 20,* 1851–1860.

Kennedy, P.J., Clarke, G., Quigley, E.M.M., Groeger, J.A., Dinan, T.G., & Cryan, J.F. (2012) Gut memories: Towards a cognitive neurobiology of irritable bowel syndrome. *Neuroscience and Biobehavioural Reviews, 36,* 310–340.

Lindfors, P., Unge, P., Nyhlin, H., Ljotsson, B., Bjornsson, E. S., Abrahamsson, H., & Simren, M. (2012). Long-term effects of hypnotherapy in patients with refractory irritable bowel syndrome. *Scandinavian Journal of Gastroenterology, 47*(4), 414-421. doi: 10.3109/00365521.2012.658858

McHorney, C. A., Ware, J. E., & Raczek, A. E. (1993). THE MOS 36-ITEM SHORT-FORM HEALTH SURVEY (SF-36) .2. PSYCHOMETRIC AND CLINICAL-TESTS OF VALIDITY IN MEASURING PHYSICAL AND MENTAL-HEALTH CONSTRUCTS. *Medical Care, 31*(3), 247-263. doi: 10.1097/00005650-199303000-00006

Naliboff, B.D., Kim, S.E., Bolus, R., Bernstein C.N., Mayer, E.A. & Chang, L. (2012). Gastrointestinal and psychological mediators of health-related quality of life in IBS

and IBD: A structural equation modeling analysis. *American Journal of Gastroenterology, 107,* (3), 451–459.
Palsson, O. S., Turner, M. J., Johnson, D. A., Burnett, C. K., & Whitehead, W. E. (2002). Hypnosis treatment for severe irritable bowel syndrome - Investigation of mechanism and effects on symptoms. *Digestive Diseases and Sciences, 47*(11), 2605-2614. doi: 10.1023/a:1020545017390
Rammstedt, B., & John, O. P. (2005). Kurzversion des Big Five Inventory (BFI-K). *Diagnostica*, *51*, 195-206.
Ruch, W., Köhler, G. & van Thriel, C. (1996). Assessing the 'humorous temperament': Construction of the facet and standard trait forms of the State-Trait-Cheerfulness-Inventory—STCI. *Humor:International Journal of Humor Research*, *9*, 303-339.
Webb, A. N., Kukuruzovic, R. H., Catto-Smith, A. G., & Sawyer, S. M. (2007). Hypnotherapy for treatment of irritable bowel syndrome. *Cochrane Database of Systematic Reviews*(4). doi: 10.1002/14651858.CD005110.pub2
Whorwell, P. J., Prior, A., & Faragher, E. B. (1984). CONTROLLED TRIAL OF HYPNOTHERAPY IN THE TREATMENT OF SEVERE REFRACTORY IRRITABLE-BOWEL SYNDROME. *Lancet, 2*(8414), 1232-1234.
Zigmond, A. S., & Snaith, R. P. (1983). THE HOSPITAL ANXIETY AND DEPRESSION SCALE. *Acta Psychiatrica Scandinavica, 67*(6), 361-370. doi: 10.1111/j.1600-0447.1983.tb09716.x

Anhang

Abb.1: Grafik zum Versuchsdesign

Anmerkung: die Fragebogenverfahren und Patienteninformationen/Einwilligungen werden aus Gründen der Übersichtlichkeit nicht hier aufgeführt, sondern als separate Dokumente eingereicht.

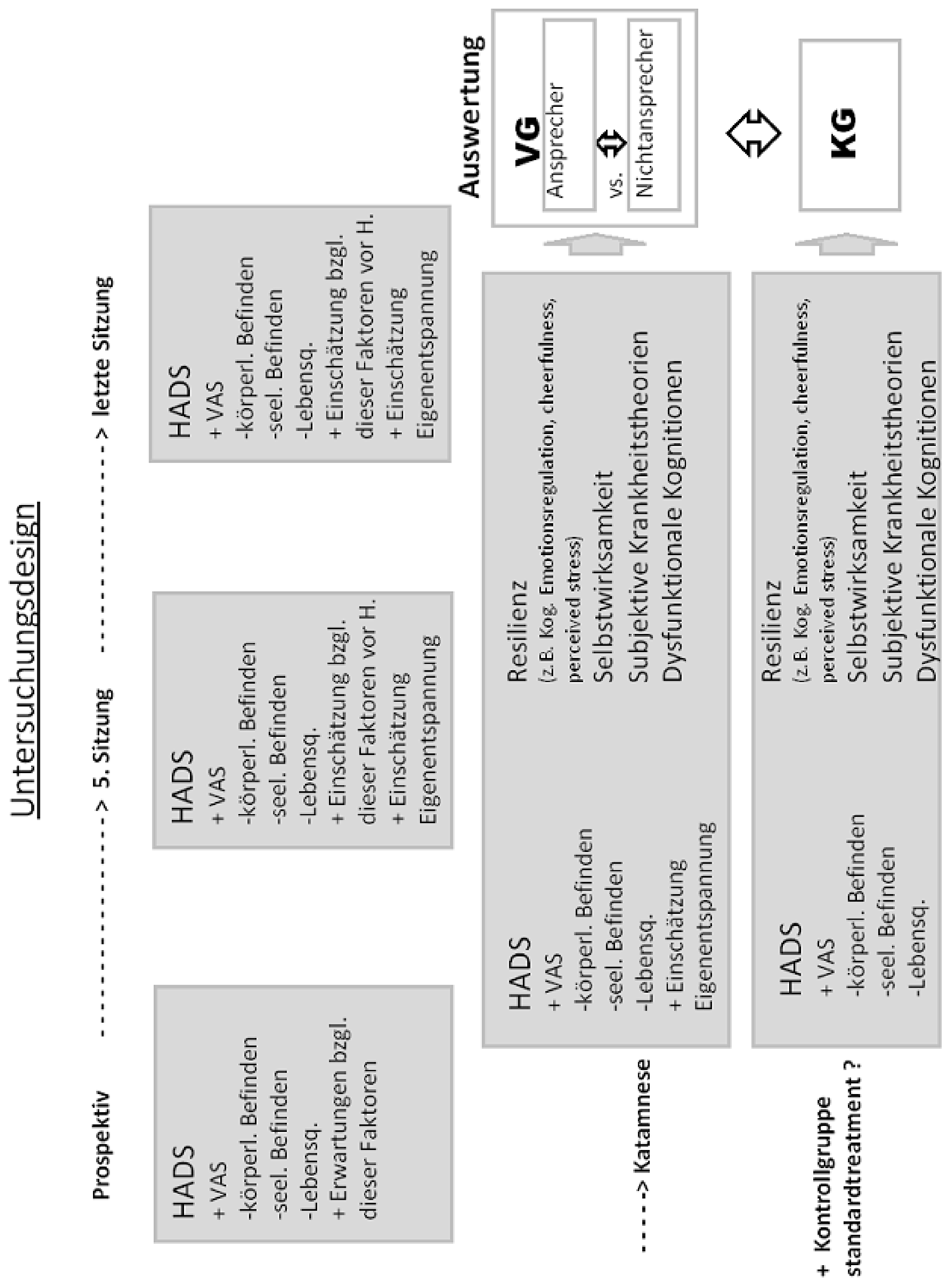

Untersuchungsdesign
Prospektiv ----------------> 5. Sitzung ----------------> letzte Sitzung
HADS
+ VAS
-körperl. Befinden
-seel. Befinden
-Lebensq.
+ Erwartungen bzgl. dieser Faktoren
HADS
+ VAS
-körperl. Befinden
-seel. Befinden
-Lebensq.
+ Einschätzung bzgl. dieser Faktoren vor H.
+ Einschätzung Eigenentspannung
HADS
+ VAS
-körperl. Befinden
-seel. Befinden
-Lebensq.
+ Einschätzung bzgl. dieser Faktoren vor H.
+ Einschätzung Eigenentspannung
-----> Katamnese
HADS
+ VAS
-körperl. Befinden
-seel. Befinden
-Lebensq.
+ Einschätzung Eigenentspannung
Resilienz
(z.B. Kog. Emotionsregulation, cheerfulness, perceived stress)
Selbstwirksamkeit
Subjektive Krankheitstheorien
Dysfunktionale Kognitionen
+ Kontrollgruppe standardtreatment ?
HADS
+ VAS
-körperl. Befinden
-seel. Befinden
-Lebensq.
Resilienz
(z.B. Kog. Emotionsregulation, cheerfulness, perceived stress)
Selbstwirksamkeit
Subjektive Krankheitstheorien
Dysfunktionale Kognitionen
Auswertung
VG
Ansprecher
vs.
Nichtansprecher
KG
