# Supplementary material for: The structure of resilience in irritable bowel syndrome and its improvement through hypnotherapy: Cross-sectional and prospective longitudinal data
Source: PLoS One. 2018 Nov 12;13(11):e0202538. doi: 10.1371/journal.pone.0202538 (PMC6231615; doi:10.1371/journal.pone.0202538)

Study protocol resilience IBS GHT Version 2.4 from the 6.8.2012

Factors of resilience in Irritable Bowel Syndrome and Gut-directed Hypnotherapy. Longitudinal study with patients in hypnotherapy with cross-sectional control group.

Dr. Ulrich Tran, University of Vienna,
Faculty of Psychology

Johannes Peter

Prof. Dr. Gabriele Moser,
Medical University of Vienna

Abstract.

Background. In Irritable Bowel Syndrome, psychological factors play an important role in illness perception and pain modulation. It is assumed that cognitive and personality factors as resilience factors might have maladaptive as well as protective influence concerning central variables of the illness. Gut-directed Hypnotherapy (GHT) is considered a worthwile therapy in irritable bowel syndrome (IBS). It is questionable, how far factors of resilience affect treatment response, and how GHT influences resilience.

Aims of the study. Insight into typical psychological characteristics of IBS. Examination of cognitive and personality factors of resilience in IBS and their change by therapy.

Methods. A study in N= 80 patients assessing the following variables: quality of life, symptom severity, anxiety and depression and measures of resilience. There are two branches: n_A= 40 patients obtained 10 sessions of GHT within routine care, which were accompanied by so far 3 assessments in variables of quality of life, and affective distress. In a catamnestic follow-up, variables of resilience and symptom severity will be assessed additionally. Respective assessments will be performed cross-sectionally in n_B= 40 patients prior to participation in GHT.

Table of contents

Abstract. 2
Table of contens 2
Project staff 3
Dr. Ulrich Tran 3
Johannes Peter 3
Univ.-Prof. Dr. Gabriele Moser 3
Authorship 3
Scientific background 3
Aims of the study 4
Study design 5
Sample 5
Inclusion criteria 6
Exclusion criteria 6
Intervention 6
Outcome variables 6
Statistics and data analysis 7
Sample size 7
Ethical considerations 8
References 8
Appendix 9

Project staff

Dr. Ulrich Tran
Institut für Psychologische Grundlagenforschung und Forschungsmethoden
Liebiggasse 5
1010 Wien
ulrich.tran@univie.ac.at

Johannes Peter
Psychology student, Universität Wien
a0609894@unet.univie.ac.at

Univ.-Prof. Dr. Gabriele Moser
Universitätsklinik für Innere Medizin III
Abteilung für Gastroenterologie und Hepatologie
Währinger Gürtel 18-20
1090 Wien
gabriele.moser@meduniwien.ac.at

Authorship

First author, Corresponding Author<br>Second author<br>Senior author	Dr. Ulrich Tran<br>Johannes Peter<br>Prof. Dr. Gabriele Moser

Scientific background

Irritable bowel syndrome (IBS) is marked by sensations of discomfort, visceral pain, and disrupted bowel emptying without known physical cause. This symptom pattern is presumably caused by dysregulations of the 'brain-gut axis' on various levels. Beside immunologic factors and alterations in the autonomous nervous system, central nervous and psychological processes may play a significant role.
Psychological aspects in IBS comprise not only high levels of distress, psychological comorbidity and heightened levels of anxiety and depression, but also variables affecting the perseption of the body. In the light of a cognitive neurobiology of IBS (Kennedy et al., 2012), cognitive and personality-related factors are crucial in the modulation of pain and the perception of central illness-related outcomes such as health-related quality of life and psychological distress. Among dysfunctional cognitions and subjective theories of illness, aspects of personality are essential as they are related to somatization, but also to protective factors of resilience.

Gut-directed Hypnotherapy (GHT) has emerged as a effective therapy in IBS (e.g. Lindfors et al., 2012; Webb, Kukuruzovic, Catto-Smith, & Sawyer, 2007; Whorwell, Prior & Faragher, 1984). The treatment protocol contains the induction of deep relaxation and trance, as well as suggestions and imaginations aiming at therapeutic change in personality, cognitive processing patterns, and illness-related attributions.

Therefore, a broad array of possible pathways of action of GHT is adressed, from cognitive and emotional change to improved autonomous nervous modulation. Numerous studies have documented inreased quality of life and lowered sensations of pain, as well as reduced psychological distress, anxiety and depression. Besides immediate cerebral improvements in pain processing (Faymonville et al., 2003), cognitive mechanisms might have a fundamental role for the efficacy of GHT. Single studies have demonstrated change in cognitive variables by GHT (Gonsalkorale, Toner & Whorwell, 2004; Palsson, Turner, Johnson, Burnett & Whitehead, 2002).

The investigation of such factors is highly relevant in the context of psychological resilience. First, because IBS-patients represent a population at high risk, since their suffering is chronic and lacks a proximate organic cause. Second, because the high comorbidity of IBS with psychological disorders brings up the question, whether these are partially based on identical mechanisms. Naliboff et al. (2012) have shown with structural equation modelling, that psychological factors have causal influence on health-related quality of life in IBS. Conversely, it is known that aspects of psychological resilience can help to retain somatic wellbeing (Ickovics et al., 2006).

Ongoing conceptualization and identification of specifically relevant factors of resilience in particular populations, as the population of IBS patients in this case, are defined goals of resilience research (Davydov, Stewart, Ritchie & Chaudieu, 2010). The present project is committed to this research objective and shall achieve a contribution to the conceptualization of resilience, the understandung of IBS, and the mechanisms of GHT.

Aims of the study

Efficacy of GHT shall be documented by change in IBS severity, quality of life, and affective variables. Concerning the captured factors of resilience, there have been no previous investigations in the population of IBS-patients at present. The study shall clarify, which factors have special relevance for illness perception and therapeutic change. Change in measures of resilience will be inferred by contrasting with the control group.

Central aims and hypotheses:

1. Decreased distress (symptom severity, affective variables, quality of life) resulting from GHT
2. Exploratory investigation of resilience variables in IBS patients
3. Resilience is associated with diminished distress perception
4. Higher resilience after GHT
5. Higher resilience in therapy responders
6. Expectation of therapy success correlates with resilience factors
7. Expectation of therapy success correlates with therapy success

Study design

Combination of a longitudinal investigation of patients in hypnotherapy and a cross-sectional investigation of patients waiting for hypnotherapy. For the longitudinal investigation, three assessments have already been performed within clinical routine (t1-t3), follow-up assessment ($t4_A$) and assessments of control group ($t4_B$) are outstanding.

A) Longitudinal recording of variables of quality of life, as well as anxiety and depression in n_A= 40 patients with IBS, who obtain GHT. Overall 4 measurements: baseline (t1), during group hypnotherapy (t2), at end of therapy (t3) and catamnesis (t4) with additional assessment of selected factors of resilience and IBS symptoms.
B) Cross-sectional assessment ($t4_B$) in n_B= 40 IBS patient without psychological treatment. Measurement in analogy to $t4_A$: variables of quality of life, anxiety and depression, factors of resilience and IBS symptoms.

For visualization of study design see appendix (Fig. 1).

The study design does not allow direct causal interpretations. The epistemic validity of the comparison between treatment and control group is supported by a relatively high stability over time of resilience and personality factors known from previous research. To assure comparability of the groups, characteristics shall be matched as far as possible.

Sample

The investigation is based on data from IBS patients of the Psychosomatic Outpatient Clinic, University Clinic, Internal Medicine III. In part can be drawn on available data from routine assessments. Approval for data recording and analysis will be applied for at the Ethikkommission der Medizinischen Universität Wien.

1. Existing data
Data from patients who obtained GHT in groups and routinely assessed between 2009 and now will be used in the study. These patients were assessed at the following points of time: prospectively before 1st session, at 5th and at 10th (last) GHT session. Captured variables were self-report data from visual analogue scales pertaining to physical, psychological, and general wellbeing. At baseline, the subjectice expectation of change in the named dimensions was asked. In the two remaining assessments, an appraisal of change relative to baseline was asked. At all points of time, HADS-D (*Hospital Anxiety and Depression scale – Deutsch*) was administered.
Questionnaires from these assessments are kept in patients' medical records at the outpatient clinic. After approval by the Ethikkommission, data from n_A= 40 patients will be reviewed, analysed statistically, and followed by a catamnestic follow-up.

2. Planned further procedure
n_A= 40 patients with existing data will be contacted by telephone and asked for permission to send further questionnaires by mail. In case of rejection, it will be asked, if GHT led to

adequate relief (to answer with yes/no). Otherwise, after given oral permission, questionnaires will be sent. Mailings will contain questionnaires, franked envelopes for sending back, as well as the patient information and the form to provide informed consent.

3. Furthermore, n_B= 40 patients will be asked to participate in the study at the outpatient clinic. After oral explanation of the study and obtained informed consent, patients will obtain questionnaires directly at the outpatient clinic.

For both subgroups it is defined:
Telephone numbers of the investigating degree candidate and the outpatient clinic (Univ. Prof. Moser) will be announced for questions. Personal support at the outpatient clinic and/or support via telephone to answer questionnaires is offered.
In case of need of additional counseling, Prof. Moser is present at the outpatient clinic or calls back.

Inclusion and exclusion criteria

**Inclusion criteria**
IBS patients after Rome-III-criteria<br>Age range: 18-70 years
**Exclusion criteria**
Pat. with acute complications<br>Pat. with severe depression or anxiety<br>Pat. with insufficient knowledge of german language<br>Pat. in pregnancy

Intervention

There is no intervention planned within the study. Half of the patients of the sample has already participated in Gut-directed Hypnotherapy. This therapy was administered at the Psychosomatic Special outpatient ambulance at the Division of Gastroenterology and Hepatology, Univ. Clinic of Internal Medicine III, 10 sessions à 45 minutes in weekly frequence, in groups of 6 zo 8 patients.
The hypnosis was administered following the *Manchester-protocol* of Gut-directed Hypnotherapy (Gonsalkorale, 2006). It can be described as follows: after a educational unit teaching cornerstones of IBS pathophysiology, a hypnotic state (relaxation/trance) is induced. Guided imagery leading to deep relaxation, imaginations and suggestions are given and hands are laid on the abdomen by the patients. This aims at dyscatastrophising of illness-related subjective beliefs and imagery, the regain of subjective control over bodily conditions, reduction of anxiety and stress, and learning of effective self-regulation.

Outcome variables

For the assessment of quality of life, affective distress, symptom severity and factors of resilience in hypnosis- and control group a number of psychological instruments will be administered.

Quality of life & affective variables
• Physical, psychological and general wellbeing (VAS= visual analogue scales)<br>• Expectation of therapy success and self-reported therapy success (only hypnosis group)<br>• Anxiety and depression (HADS-D, Zigmond & Snaith, 1983)
**Symptom Severity**
• IBS-Symptom Severity Scale, German translation<br>• Question for adequate relief, dichotomous answering
**Resilience factors**
• BFI-K Big Five Personality, factors extraversion und neuroticism (Rammstedt & John, 2005)<br>• State-Trait Cheerfulness Inventory (Ruch, Köhler, & van Thriel, 1996)<br>• SWE Self-efficacy (Schwarzer & Jerusalem, 1999)<br>• CD-RISC Resilience Scale brief version (Connor & Davidson, 2003)<br>• CERQ cognitive emotion regulation (Loch, Hiller, & Witthöft, 2011)<br>• F-SozU K-14 Social Support (Fydrich, Sommer, Tydecks,& Brähler, 2009)

Statistics and data analysis

Data from questionnaires and medical records will be assigned codes, electronic processing will be anonymously. Statistical analyises will be performed with SPSS (version 16 or higher). Analysis of obtainied data will be descriptive, and inferential for the most important hypotheses (change in quality of life, anxiety and depression after GHT). Planned are multiple regression, Pearson correlations, factorial analysis of variance, and multivariate analysis of variance for repeated measures (repeated-measures MANOVAs) fort he longitudinal analyses of the hypnosis group.

Sample size

In a recently performed study by Prof. Moser at the study site (general hospital of Vienna) in IBS patients and Gut-directed Hypnotherapy (submitted for publication), in the SF-36 questionnaire pertaining to health-related quality of life (McHorney, Ware & Raczek, 1993) occured differences with effect sizes of d= .34 on average; with the program G*Power 3.1 (Faul, Erdfelder, Buchner & Lang, 2009) it was calculated, that a sample size of 49 persons per group is required at a alpha-error level of 5 % and at a power of .80. Flik et al. (2011) have publicated a study protocol and calculated a group size of 44 for a valid catamnestic contrasting of patients who obtained GHT and placebo controls. The present study is approximately fulfilling these requirements with 40 persons per group.

Ethical considerations

For the patients, participation is requires a certain amount of time to fill out the questionnaires. The time spent and reporting intimate attitudes can be perceived as adverse in some circumstances. On the other hand, it is positive to contribute to clarification of one‘s own disease and to common scientific goals. There is no known risk for health associated with the answering of questionnaires.

References

Connor, K. M., & Davidson, J. R. T. (2003). Development of a new resilience scale: The Connor–Davidson Resilience Scale (CDRISC). *Depression and Anxiety*, *18*, 76-82.

Davydov, D.M., Stewart, R., Ritchie, K., & Chaudieu, I. (2010). Resilience and mental health. *Clinical Psychology Review, 30,* 479–495.

Faul, F., Erdfelder, E., Buchner, A., & Lang, A.-G. (2009). Statistical power analyses using G*Power 3.1: Tests for correlation and regression analyses. *Behavior Research Methods, 41*, 1149–1160.

Faymonville, M. E., Roediger, L., Del Fiore, G., Delgueldre, C., Phillips, C., Lamy, M., . . . Laureys, S. (2003). Increased cerebral functional connectivity underlying the antinociceptive effects of hypnosis. *Cognitive Brain Research, 17*(2), 255-262. doi: 10.1016/s0926-6410(03)00113-7

Flik, C.E., van Rood, Y.R., Laan, W., Smout, A.J., Weusten, B.L., Whorwell, P.J., & de Wit, N.J. (2011). A randomised controlled trial on hypnotherapy for irritable bowel syndrome: design and methodological challenges (the IMAGINE study). *Gastroenterology, 11*(137), 1–10.

Francis, C.Y., Morris, J., & Whorwell PJ. (1997). The irritable bowel severity scoring system: a simple method of monitoring irritable bowel syndrome and its progress. *Alimentary Pharmacology & Therapeutics, 11*, 395–402. doi: 10.1046/j.1365-2036.1997.142318000.x

Fydrich, T., Sommer, G., Tydecks, S., & Brähler, E. (2009). Fragebogen zur sozialen Unterstützung (FSozU):Normierung der Kurzform (K-14). *Zeitschrift für Medizinische Psychologie*, *18*, 43-48.

Gonsalkorale, W. M. (2006). Gut-directed hypnotherapy: The Manchester approach for treatment of irritable bowel syndrome. *International Journal of Clinical and Experimental Hypnosis, 54*(1), 27-50. doi: 10.1080/00207140500323030

Gonsalkorale, W. M., Toner, B. B., & Whorwell, P. J. (2004). Cognitive change in patients undergoing hypnotherapy for irritable bowel syndrome. *Journal of Psychosomatic Research, 56*(3), 271-278. doi: 10.1016/s0022-3999(03)00076-x

Ickovics, J.R., Milan, S., Boland, R., Schoenbaum, E., Schuman, P., & Vlahov, D. (2006). Psychological resources protect health: 5-year survival and immune function among HIV-infected women from four US cities. *AIDS, 20,* 1851–1860.

Kennedy, P.J., Clarke, G., Quigley, E.M.M., Groeger, J.A., Dinan, T.G., & Cryan, J.F. (2012) Gut memories: Towards a cognitive neurobiology of irritable bowel syndrome. *Neuroscience and Biobehavioural Reviews, 36,* 310–340.

Lindfors, P., Unge, P., Nyhlin, H., Ljotsson, B., Bjornsson, E. S., Abrahamsson, H., & Simren, M. (2012). Long-term effects of hypnotherapy in patients with refractory irritable bowel syndrome. *Scandinavian Journal of Gastroenterology, 47*(4), 414-421. doi: 10.3109/00365521.2012.658858

McHorney, C. A., Ware, J. E., & Raczek, A. E. (1993). THE MOS 36-ITEM SHORT-FORM HEALTH SURVEY (SF-36) .2. PSYCHOMETRIC AND CLINICAL-TESTS OF VALIDITY IN MEASURING PHYSICAL AND MENTAL-HEALTH CONSTRUCTS. *Medical Care, 31*(3), 247-263. doi: 10.1097/00005650-199303000-00006

Naliboff, B.D., Kim, S.E., Bolus, R., Bernstein C.N., Mayer, E.A. & Chang, L. (2012). Gastrointestinal and psychological mediators of health-related quality of life in IBS and IBD: A structural equation modeling analysis. *American Journal of Gastroenterology, 107,* (3), 451–459.

Palsson, O. S., Turner, M. J., Johnson, D. A., Burnett, C. K., & Whitehead, W. E. (2002). Hypnosis treatment for severe irritable bowel syndrome - Investigation of mechanism and effects on symptoms. *Digestive Diseases and Sciences, 47*(11), 2605-2614. doi: 10.1023/a:1020545017390

Rammstedt, B., & John, O. P. (2005). Kurzversion des Big Five Inventory (BFI-K). *Diagnostica*, *51*, 195-206.

Ruch, W., Köhler, G. & van Thriel, C. (1996). Assessing the 'humorous temperament': Construction of the facet and standard trait forms of the State-Trait-Cheerfulness-Inventory—STCI. *Humor:International Journal of Humor Research*, *9*, 303-339.

Webb, A. N., Kukuruzovic, R. H., Catto-Smith, A. G., & Sawyer, S. M. (2007). Hypnotherapy for treatment of irritable bowel syndrome. *Cochrane Database of Systematic Reviews*(4). doi: 10.1002/14651858.CD005110.pub2

Whorwell, P. J., Prior, A., & Faragher, E. B. (1984). CONTROLLED TRIAL OF HYPNOTHERAPY IN THE TREATMENT OF SEVERE REFRACTORY IRRITABLE-BOWEL SYNDROME. *Lancet, 2*(8414), 1232-1234.

Zigmond, A. S., & Snaith, R. P. (1983). THE HOSPITAL ANXIETY AND DEPRESSION SCALE. *Acta Psychiatrica Scandinavica, 67*(6), 361-370. doi: 10.1111/j.1600-0447.1983.tb09716.x

Appendix

Fig 1: Depiction of study design

Annotation: Questionnaires and patient information / consent form are not listed here, but are submitted separately.

Untersuchungsdesign

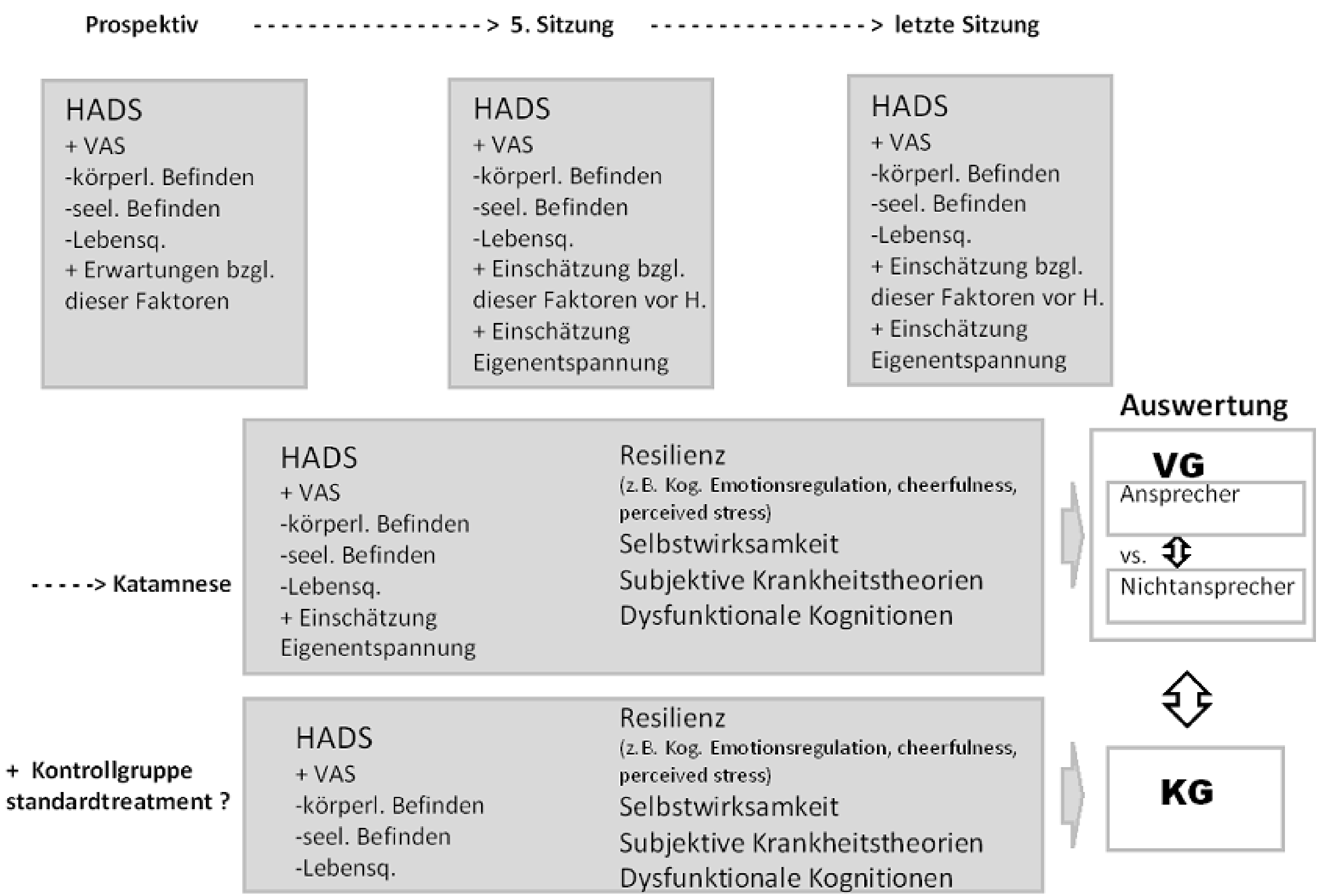

Supplement: S3 Appendix — (PDF) [file pone.0202538.s003.pdf]
